# Immunomodulatory effects of ulinastatin combined with continuous blood purification in sepsis: a systematic review and meta-analysis

**DOI:** 10.3389/fphar.2025.1591470

**Published:** 2025-07-01

**Authors:** Hui Gao, Zhiqiang Tang, Shucheng Zhao, Yuanjun Zhang

**Affiliations:** ^1^ Hepatobiliary and Pancreatic Surgery Department, Ziyang Central Hospital, Ziyang, China; ^2^ Emergency Department, Ziyang Central Hospital, Ziyang, China; ^3^ Intensive Care and Emergency Medicine Department, Ziyang Central Hospital, Ziyang, China

**Keywords:** ulinastatin, continuous blood purification, sepsis, inflammatory markers, meta-analysis

## Abstract

**Background:**

Sepsis involves a dysregulated immune response to infection, causing inflammation and organ dysfunction. This systematic review and meta-analysis evaluated the immunomodulatory effects of ulinastatin combined with continuous blood purification (CBP) in sepsis.

**Methods:**

This study involved a literature search, data extraction, quality assessment, and meta-analysis to evaluate the effects of ulinastatin combined with CBP. A total of 34 studies, including 28 randomized controlled trials (RCTs) and 6 retrospective studies involving patients with sepsis, were included.

**Results:**

The pooled results demonstrated significant reductions in inflammatory markers including CRP (SMD: 2.210, 95% CI: 2.760 to −1.661, P < 0.0001), IL-1β (SMD: 1.536, 95% CI: 1.773 to −1.299, P < 0.0001), IL-6 (SMD: 2.679, 95% CI: 3.271 to −2.086, P < 0.0001), IL-8 (SMD: 2.959, 95% CI: 4.582 to −1.337, P < 0.0001), IL-10 (SMD: 4.449, 95% CI: 7.216 to −1.682, P = 0.002), PCT (SMD: 3.787, 95% CI: 4.597 to −2.977, P < 0.0001), TNF-α (SMD: 2.734, 95% CI: 3.480 to −1.987, P < 0.0001), and mortality (OR: 0.30, 95% CI: 0.22 to 0.42, P < 0.0001) in ulinastatin group compared with control group. Egger’s test indicated significant publication bias (P = 0.002).

**Conclusion:**

Ulinastatin combined with CBP significantly reduces inflammatory markers and mortality in sepsis patients, suggesting its potential benefit in managing sepsis-related inflammation. Further studies are needed to confirm these findings.

## 1 Introduction

Sepsis is a severe and potentially life-threatening condition resulting from the body’s overwhelming and dysregulated response to infection (Rello et al., 2017). Sepsis is frequently encountered in the Intensive Care Unit (ICU) due to its severe nature and the need for intensive monitoring and treatment. Patients in the ICU are often critically ill and may develop sepsis as a complication of their underlying conditions or hospital-acquired infections (Fleischmann-Struzek et al., 2020). The incidence of sepsis has significantly increased over the past decades, with an annual rise of about 1.5%–8%, making it a major health concern worldwide (Patel et al., 2007). It can lead to tissue damage, organ failure, and death if not promptly recognized and treated. Sepsis can be triggered by infections from bacteria, viruses, fungi, or parasites, affecting any part of the body, including the lungs, urinary tract, abdomen, and skin (Huang et al., 2019). Current treatment for sepsis focuses on limiting the development of organ dysfunction by providing rapid control of infection, hemodynamic stabilization, and organ support to ensure recovery of organ function (Lelubre and Vincent, 2018). Despite significant advancements in anti-infective therapy and organ function support techniques, some patients still struggle to control the body’s inflammatory response, leading to dysfunction or failure of organs such as the heart, liver, and kidneys (Gustot et al., 2009). Therefore, improving the inflammatory state and protecting organs in patients with sepsis is of crucial clinical significance.

Ulinastatin is a broad-spectrum protease inhibitor derived from human urine. It has potent inhibitory effects on various endogenous proteases such as trypsin, chymotrypsin, and elastase, making it a valuable therapeutic agent in the treatment of several critical conditions, particularly those involving severe inflammation and organ dysfunction (Umeadi et al., 2008). Ulinastatin has been shown to be effective in reducing inflammation and improving clinical outcomes in patients with acute pancreatitis. It reduces enzyme levels and inflammation markers, leading to faster recovery and reduced severity of the condition (Chen et al., 2017). In cases of severe sepsis and acute circulatory failure, ulinastatin has demonstrated efficacy in controlling systemic inflammation and improving patient outcomes. Its ability to inhibit inflammatory mediators helps in stabilizing hemodynamics and reducing organ damage (Wang et al., 2013). Xu et al. reported that ulinastatin combined with continuous blood purification (CBP) significantly reduces inflammation, oxidative stress, and myocardial injury while enhancing immune function in septic shock patients, showing high clinical value (He et al., 2021). Ni et al. investigated the therapeutic effects of combining ulinastatin with CBP in treating severe sepsis, and ICU patients were divided into two groups including one receiving only CBP and the other receiving CBP plus ulinastatin. The study suggested that post-treatment levels of inflammatory mediators Interleukin-6 (IL-6) and Tumor Necrosis Factor-alpha (TNF-α), as well as D-dimer and PCT, were significantly lower in the control group. This combination therapy effectively reduces inflammation and improves survival rates, demonstrating notable therapeutic benefits (Ni et al., 2019). To date, there has been no comprehensive systematic review reporting on the immunomodulatory effects of ulinastatin in the treatment of sepsis. This highlights the necessity of conducting a systematic review and meta-analysis to provide a consolidated and evidence-based understanding of the therapeutic efficacy and immunomodulatory mechanisms of ulinastatin in sepsis management.

Therefore, this systematic review and meta-analysis aimed to evaluate the immunomodulatory effects of ulinastatin combined with CBP in sepsis.

## 2 Materials and methods

This systematic review and meta-analysis was conducted followed the Preferred Reporting Items for Systematic Reviews and Meta-Analyses (PRISMA) guidelines to assess immunomodulatory effects of ulinastatin for sepsis (Moher et al., 2009). The review protocol was registered with International Prospective Register of Systematic Reviews (PROSPERO).

### 2.1 Search strategy

A comprehensive literature search was performed across multiple databases, including PubMed, Embase, Cochrane Library, Web of Science, CNKI, WanFang, and Sinomed, up to 3 May 2024. The search strategy included a broad range of terms related to ulinastatin and sepsis to capture relevant studies without language or publication date restrictions. The search strategy combined terms related to “bloodstream infection,” “inflammation,” and “urinary trypsin inhibitor.” Keywords and MeSH terms included “bloodstream infection,” “sepsis,” “inflammation,” “ulinastatin,” and relevant variations. The detailed search strategy for each database was summarized in Supplementary Table S1.

### 2.2 Inclusion and exclusion criteria

The inclusion and exclusion criteria were as follows: The inclusion criteria included (Rello et al., 2017) studies reporting patients diagnosed with sepsis (Fleischmann-Struzek et al., 2020); patients receiving treatment with ulinastatin combined with CBP therapy (Patel et al., 2007); studies including conventional therapy or CBP therapy alone as a comparator (Huang et al., 2019); outcome measures including levels of inflammatory markers such as Interleukin-1 beta (IL-1β), IL-6, Interleukin-8 (IL-8), Interleukin-10 (IL-10), C-reactive protein (CRP), Procalcitonin (PCT), and TNF-α. The exclusion criteria were as follows (Rello et al., 2017): reviews, conference papers, and study designs (Fleischmann-Struzek et al., 2020); single-arm studies (Patel et al., 2007); duplicate studies (Huang et al., 2019); studies with non-extractable data.

### 2.3 Study selection

The study selection process was conducted independently by two reviewers. Initially, the reviewers screened the titles and abstracts of all identified records to determine their relevance. Studies that appeared potentially eligible based on this preliminary screening were then subjected to a full-text assessment to confirm their eligibility for inclusion. Any discrepancies between the two reviewers were resolved through discussion or by consulting a third reviewer to ensure accuracy and consistency in the selection process.

### 2.4 Data extraction and quality assessment

Data extraction was conducted independently by two reviewers. Relevant information was extracted from each study, including study design, sample size, patient demographics, interventions of experimental and control groups, test methods, and outcomes of interest. The quality assessment of the included studies was performed using the Cochrane Risk of Bias Tool (Higgins et al., 2011). To ensure consistency and accuracy, any discrepancies between the two reviewers were resolved through discussion or by consulting a third reviewer.

### 2.5 Data analysis

Data analysis for this meta-analysis was conducted using Stata version 12.0. Standardized Mean Differences (SMD) along with their corresponding 95% confidence intervals (CI) were calculated for continuous outcomes across studies. Heterogeneity among the studies was assessed using the I^2^ statistic, with values of 25%, 50%, and 75% representing low, moderate, and high heterogeneity, respectively. Based on the level of heterogeneity, either a fixed-effects model or a random-effects model was applied. Specifically, a fixed-effects model was used when heterogeneity was low (I^2^ < 50%), and a random-effects model was used when heterogeneity was high (I^2^ ≥ 50%). Subgroup analyses were performed based on the study design and doses of ulinastatin to explore potential sources of heterogeneity and to determine the robustness of the overall findings. Sensitivity analyses were conducted by excluding each study one by one to assess the stability of the results. Publication bias was evaluated visually using funnel plots and statistically using Egger’s test. A funnel plot was generated to detect asymmetry, and Egger’s test was used to assess the statistical significance of the publication bias (Egger et al., 1997).

## 3 Results

### 3.1 Study selection

**FIGURE 1:**
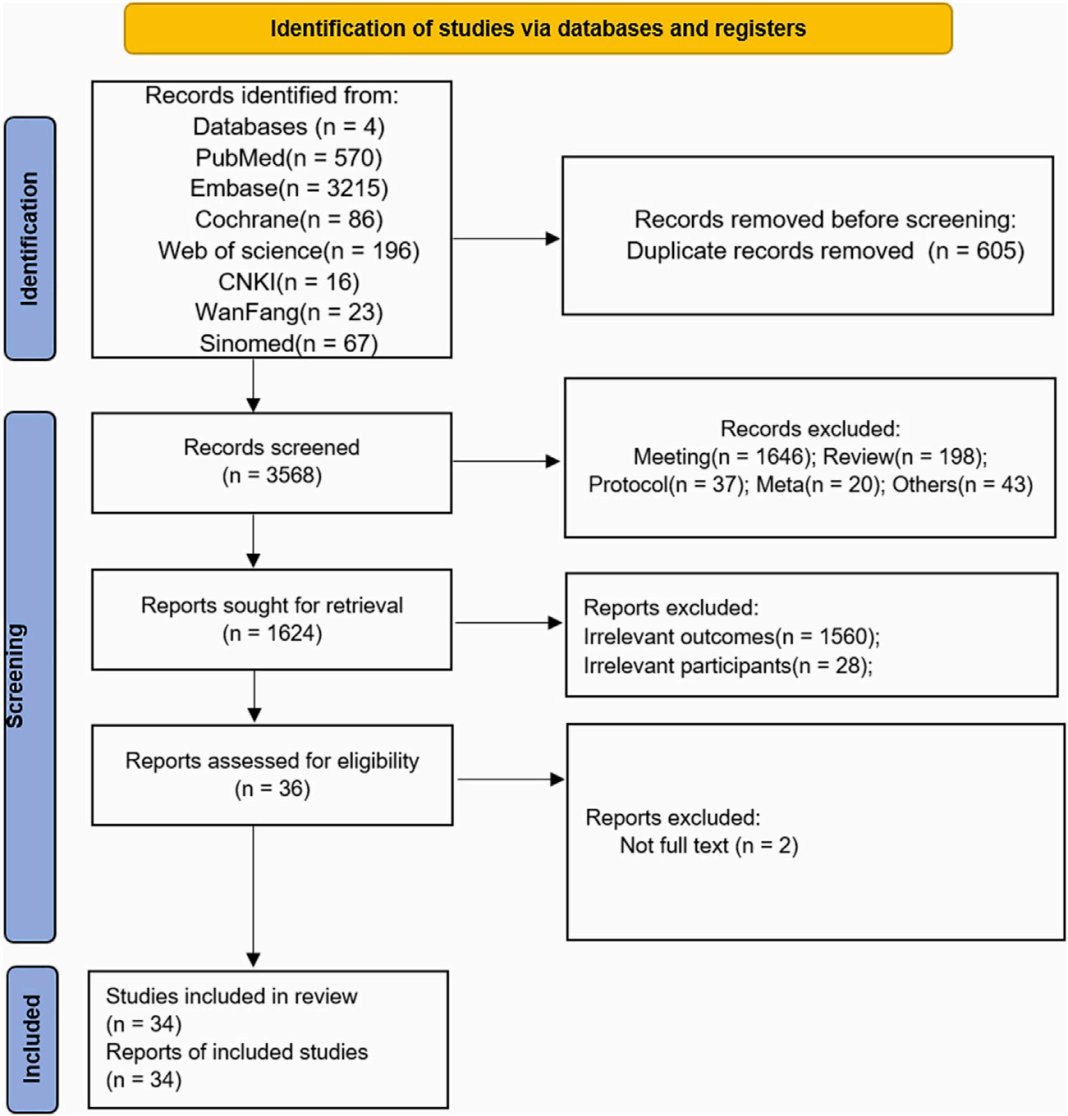
PRISMA study selection Flow Diagram. PRISMA, Preferred reporting items for systematic reviews and meta-analyses.

As illustrated in Figure 1, the comprehensive search across multiple databases and registers, including PubMed, Embase, Cochrane, Web of Science, CNKI, WanFang, and Sinomed, identified a total of 3,968 records. Following the removal of 605 duplicate records, 3,568 records were screened based on their titles and abstracts. The initial screening phase excluded 2,001 records for reasons such as meeting abstracts (n = 1,646), review articles (n = 198), study protocols (n = 37), meta-analyses (n = 20), and other unrelated documents (n = 43), resulting in 1,624 reports being selected for full-text assessment. Upon detailed review, 1,588 reports were further excluded due to irrelevant outcomes (n = 1,560), irrelevant participants (n = 28), or lack of full-text availability (n = 2). This left 36 reports that were assessed for eligibility. Ultimately, 34 studies met the inclusion criteria and were included in the final review (Figure 1).

### 3.2 Characteristics of included studies

This meta-analysis included data from 34 studies investigating the use of ulinastatin combined with continuous blood purification (CBP) therapy in patients with sepsis, conducted between 2016 and 2024 (Table 1) (He et al., 2021; Ni et al., 2019; Yao and Yuan, 2020; Feng, 2020; Sun, 2020; Zeng, 2018; Zhang, 2020; Chao et al., 2016; Fang and Zhao, 2017; Wen and Chen, 2023; Li et al., 2020; Wei et al., 2022; Cen et al., 2021; Ma, 2019; Qiu et al., 2017; Bao, 2019; Lian, 2023; Li, 2021; Li et al., 2018; Xu and Fu, 2021; Xu et al., 2024; Zhao and Chen, 2019; Qiao and Shang, 2018; Dong et al., 2023; Dong and Feng, 2023; Li et al., 2024; Xia, 2022; Zhou, 2021; Jin, 2019; Jin et al., 2021; Fang et al., 2018; Lei et al., 2023; Sun, 2021; Lv et al., 2020). The studies consisted of 28 randomized controlled trials (RCTs) and 6 retrospective studies. All studies were conducted in China. The sample sizes varied across studies, ranging from 20 to 120 patients in the experimental groups and 20 to 120 patients in the control groups. The age of participants spanned from 33.99 to 71.4 years, with the percentage of female participants ranging from 30% to 54.17%. Interventions in the experimental groups involved intravenous injections of ulinastatin at varying doses, combined with continuous blood purification therapy, while the control groups received continuous blood purification therapy alone. The duration of treatment varied from 3 days to 10 days. The studies employed different test methods, including ELISA and turbidimetric inhibition immunoassays, to measure outcomes. The primary outcomes assessed were levels of inflammatory markers such as IL-1β, IL-6, IL-8, IL-10, CRP, PCT, and TNF-α, providing a comprehensive overview of the effectiveness of the combined therapy on inflammatory responses in sepsis patients. The detailed characteristics of each study are summarized in Table 1. The included studies, assessed using the Cochrane Risk of Bias Tool, exhibited minimal risk of bias across various domains, indicating a high level of methodological rigor and reliability (Supplementary Figures S1, S2).

**TABLE 1:** Characteristic of the included studies.

Study	Country	Study design	Sample size (e.g.,/cg)	Age	Female%	Intervention of, e.g.,	Intervention of cg	Duration of treatment	Comparisons	Test methods	Outcomes
Fang and Zhao (2017)	China	RCT	49/47	57.97	32.29	intravenous injection of 300,000 U of ulinastatin + CBP	CBP	5 days	ulinastatin + CBP vs. CBP	ELISA	PCT, CRP, TNF-a, IL-6
Ni et al. (2019)	China	RCT	60/60	56	31.67	intravenous injection of 200,000 U of ulinastatin + CBP	CBP	1 week	ulinastatin + CBP vs. CBP	\	IL-6, TNF-a, PCT
Li et al. (2024)	China	RCT	120/120	53.13	54.17	intravenous injection of 200,000 U of ulinastatin + CBP	CBP	1 week	ulinastatin + CBP vs. CBP	\	PCT
Lv et al. (2020)	China	RCT	50/50	5.25	44	intravenous injection of 20,000 U/kg of ulinastatin + CBP	CBP	1 week	ulinastatin + CBP vs. CBP	ELISA	CRP, PCT
Dong et al. (2023)	China	retrospective study	28/28	51.68	42.86	intravenous injection of 200,000 U of ulinastatin + CBP	CBP	1 week	ulinastatin + CBP vs. CBP	\	PCT
Ma (2019)	China	RCT	34/34	45.06	45.59	intravenous injection of 200,000 U of ulinastatin + CBP	CBP	1 week	ulinastatin + CBP vs. CBP	ELISA	PCT
Li et al. (2020)	China	RCT	60/60	60.8	39.17	intravenous injection of 500,000 U of ulinastatin + CBP	CBP	1 week	ulinastatin + CBP vs. CBP	ELISA	PCT, CRP, IL-10
Yao and Yuan (2020)	China	RCT	32/32	45.26	40.63	intravenous injection of 200,000 U of ulinastatin + CBP	CBP	1 week	ulinastatin + CBP vs. CBP	\	PCT
Yao and Yuan (2020)	China	RCT	30/30	60.12	40	intravenous injection of 300,000 U of ulinastatin + CBP	CBP	5 days	ulinastatin + CBP vs. CBP	ELISA	CRP, TNF-a, IL-6
Fang et al. (2018)	China	RCT	40/40	55.45	36.25	intravenous injection of 500,000 U of ulinastatin + CBP	CBP	1 week	ulinastatin + CBP vs. CBP	ELISA	CRP, TNF-a, IL-6
Qiu et al. (2017)	China	RCT	30/30	44.25	35	intravenous injection of 200,000 U of ulinastatin + CBP	CBP	1 week	ulinastatin + CBP vs. CBP	ELISA	TNF-a, IL-6
Qiao and Shang (2018)	China	RCT	50/50	53.27	44	intravenous injection of 200,000 U of ulinastatin + CBP	CBP	1 week	ulinastatin + CBP vs. CBP	Turbidimetric inhibition immuno assay	PCT, CRP
Zeng (2018)	China	retrospective study	45/35	52.54	36.25	intravenous injection of 300,000 U of ulinastatin + CBP	CBP	5 days	ulinastatin + CBP vs. CBP	ELISA	TNF-a, IL-6, IL-8
Sun (2020)	China	RCT	20/20	52.24	37.5	intravenous injection of 200,000 U of ulinastatin + CBP	CBP	1 week	ulinastatin + CBP vs. CBP	ELISA	TNF-a, IL-6, PCT,CRP
Zhou (2021)	China	RCT	41/41	53.85	45.12	intravenous injection of 200,000 U of ulinastatin + CBP	CBP	1 week	ulinastatin + CBP vs. CBP	ELISA	IL-10, CRP
Wen and Chen (2023)	China	retrospective study	35/35	58.69	47.14	intravenous injection of 100,000 U of ulinastatin + CBP	CBP	1 week	ulinastatin + CBP vs. CBP	Turbidimetric inhibition immuno assay	TNF-a, IL-6, PCT,CRP,IL-10
Feng (2020)	China	RCT	34/34	33.99	47.06	intravenous injection of 200,000 U of ulinastatin + CBP	CBP	10 days	ulinastatin + CBP vs. CBP	\	TNF-a, IL-6
Li (2021)	China	RCT	35/35	49.64	47.14	intravenous injection of 200,000 U of ulinastatin + CBP	CBP	1 week	ulinastatin + CBP vs. CBP	\	PCT
Li et al. (2018)	China	RCT	40/40	55.9	31.25	intravenous injection of 200,000 U of ulinastatin + CBP	CBP	1 week	ulinastatin + CBP vs. CBP	\	PCT
Ming 2020	China	RCT	28/28	47.1	37.5	intravenous injection of 300,000 U of ulinastatin + CBP	CBP	10 days	ulinastatin + CBP vs. CBP	\	TNF-a, IL-6, IL-1b
Kui 2016	China	RCT	20/20	52.32	30	intravenous injection of 300,000 U of ulinastatin + CBP	CBP	10 days	ulinastatin + CBP vs. CBP	\	TNF-a, IL-6
Xu and Fu (2021)	China	RCT	71/71	68.99	46.48	intravenous injection of 100,000 U of ulinastatin + CBP	CBP	1 week	ulinastatin + CBP vs. CBP	ELISA	TNF-a, IL-6, PCT,CRP
Dong and Feng (2023)	China	RCT	62/62	51.99	46.77	intravenous injection of 200,000 U of ulinastatin + CBP	CBP	1 week	ulinastatin + CBP vs. CBP	ELISA	CRP, PCT
Lian (2023)	China	retrospective study	23/23	71.4	52.17	intravenous injection of 200,000 U of ulinastatin + CBP	CBP	5 days	ulinastatin + CBP vs. CBP	ELISA	CRP
Xu et al. (2024)	China	RCT	51/51	55.59	40.2	intravenous injection of 500,000 U of ulinastatin + CBP	CBP	1 week	ulinastatin + CBP vs. CBP	\	PCT,CRP,IL-10
He et al. (2021)	China	RCT	25/23	53.32	37.5	intravenous injection of 200,000 U of ulinastatin + CBP	CBP	1 week	ulinastatin + CBP vs. CBP	ELISA	CRP
Sun (2021)	China	RCT	55/55	51.1	40.91	intravenous injection of 200,000 U of ulinastatin + CBP	CBP	\	ulinastatin + CBP vs. CBP	\	CRP
Bao (2019)	China	RCT	38/38	61.04	42.11	intravenous injection of 200,000 U of ulinastatin + CBP	CBP	3 days	ulinastatin + CBP vs. CBP	ELISA	TNF-a, IL-6, IL-8
Lei et al. (2023)	China	RCT	34/34	45.67	42.65	intravenous injection of 200,000 U of ulinastatin + CBP	CBP	1 week	ulinastatin + CBP vs. CBP	\	TNF-a, IL-6, IL-1b, CRP
Jin et al. (2021)	China	RCT	30/30	52.67	48.33	intravenous injection of 200,000 U of ulinastatin + CBP	CBP	1 week	ulinastatin + CBP vs. CBP	\	CRP, PCT
Xia (2022)	China	RCT	40/40	65.6	46.25	intravenous injection of 200,000 U of ulinastatin + CBP	CBP	1 week	ulinastatin + CBP vs. CBP	\	PCT
Jin (2019)	China	RCT	44/44	58.31	38.64	intravenous injection of 200,000 U of ulinastatin + CBP	CBP	3 days	ulinastatin + CBP vs. CBP	ELISA	TNF-a, IL-6
Zhao and Chen (2019)	China	retrospective study	28/28	37.25	48.21	intravenous injection of 200,000 U of ulinastatin + CBP	CBP	5 days	ulinastatin + CBP vs. CBP	immunochemiluminescence	TNF-a, IL-6, IL-1b
Wei et al. (2022)	China	retrospective study	100/80	54.68	30.56	intravenous injection of 200,000 U of ulinastatin + CBP	CBP	1 week	ulinastatin + CBP vs. CBP	\	CRP, IL-1b

Abbreviations: U, units; CBP, continuous blood purification; CRP, C-reactive protein; PCT, procalcitonin; TNF-α, tumor necrosis factor-alpha; IL, interleukin; ELISA, enzyme-linked immunosorbent assay.

### 3.3 Meta-analysis of CRP

**FIGURE 2:**
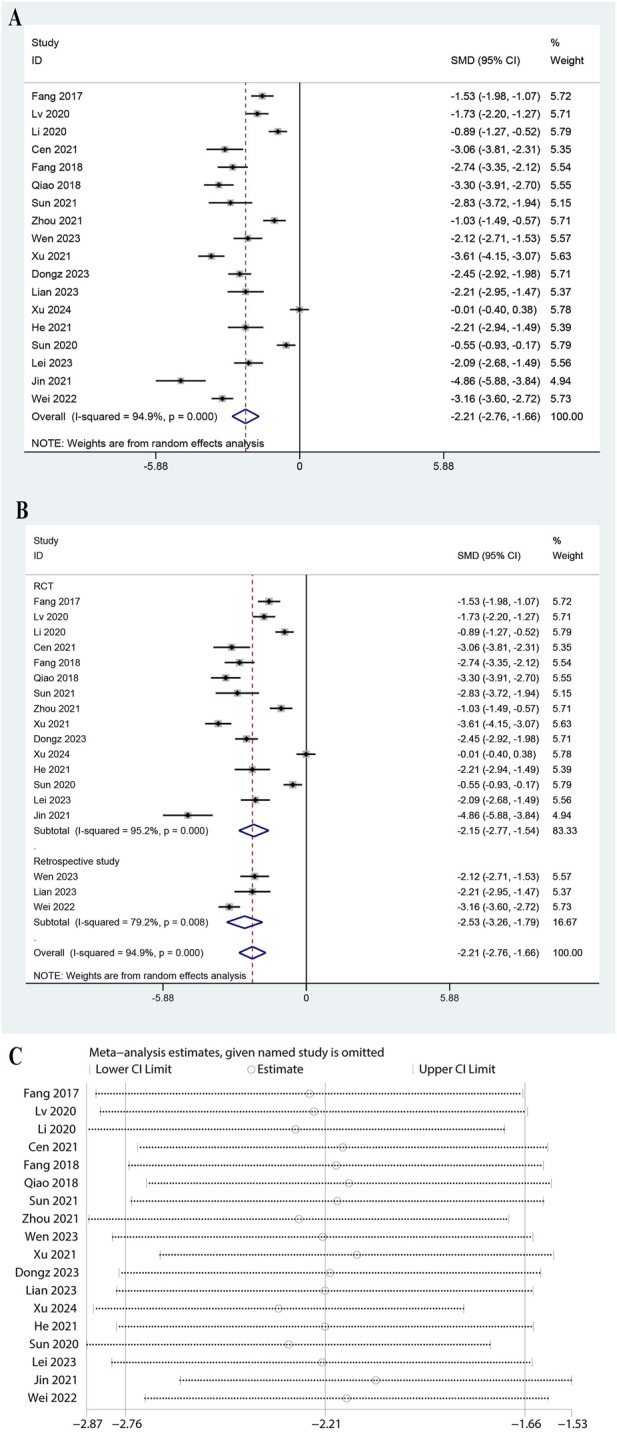
Meta-analysis of Effect of Ulinastatin Combined with Continuous Blood Purification versus Control on C-Reactive Protein (CRP) for Patients with Sepsis. **(A)**: Forest plot displays the pooled standardized mean differences (SMD) with 95% confidence intervals (CI) for the effect. **(B)**: Subgroup analysis shows separate meta-analyses stratified by study design. **(C)**: Sensitivity analysis illustrates leave-one-out analyses to evaluate the influence of individual studies on the overall effect size.

The meta-analysis of 18 studies showed that ulinastatin combined with CBP significantly reduced CRP levels compared to the control group. The pooled Standardized Mean Difference (SMD) for CRP indicated a substantial reduction (SMD: 2.210, 95% CI: 2.760 to −1.661, P < 0.0001, Figure 2A). Subgroup analysis revealed consistent findings for both randomized controlled trials (RCTs) and retrospective studies, with high heterogeneity in the RCT subgroup (I^2^ = 95.2%, P = 0.000) and moderate heterogeneity in the retrospective studies (Figure 2B, I^2^ = 79.2%, P = 0.008). Subgroup analysis for doses of ulinastatin showed a significant reduction in both the >200,000 U group (SMD: 1.81, 95% CI: 3.21 to −0.42, P < 0.05, I^2^ = 96.5%) and the ≤200,000 U group (SMD: 2.33, 95% CI: 2.92 to −1.73, P < 0.05, I^2^ = 94.2%, Supplementary Figure S3). The sensitivity analysis demonstrated that the overall results remained stable when any single study was omitted from the meta-analysis (Figure 2C).

### 3.4 Meta-analysis of IL-1β

**FIGURE 3:**
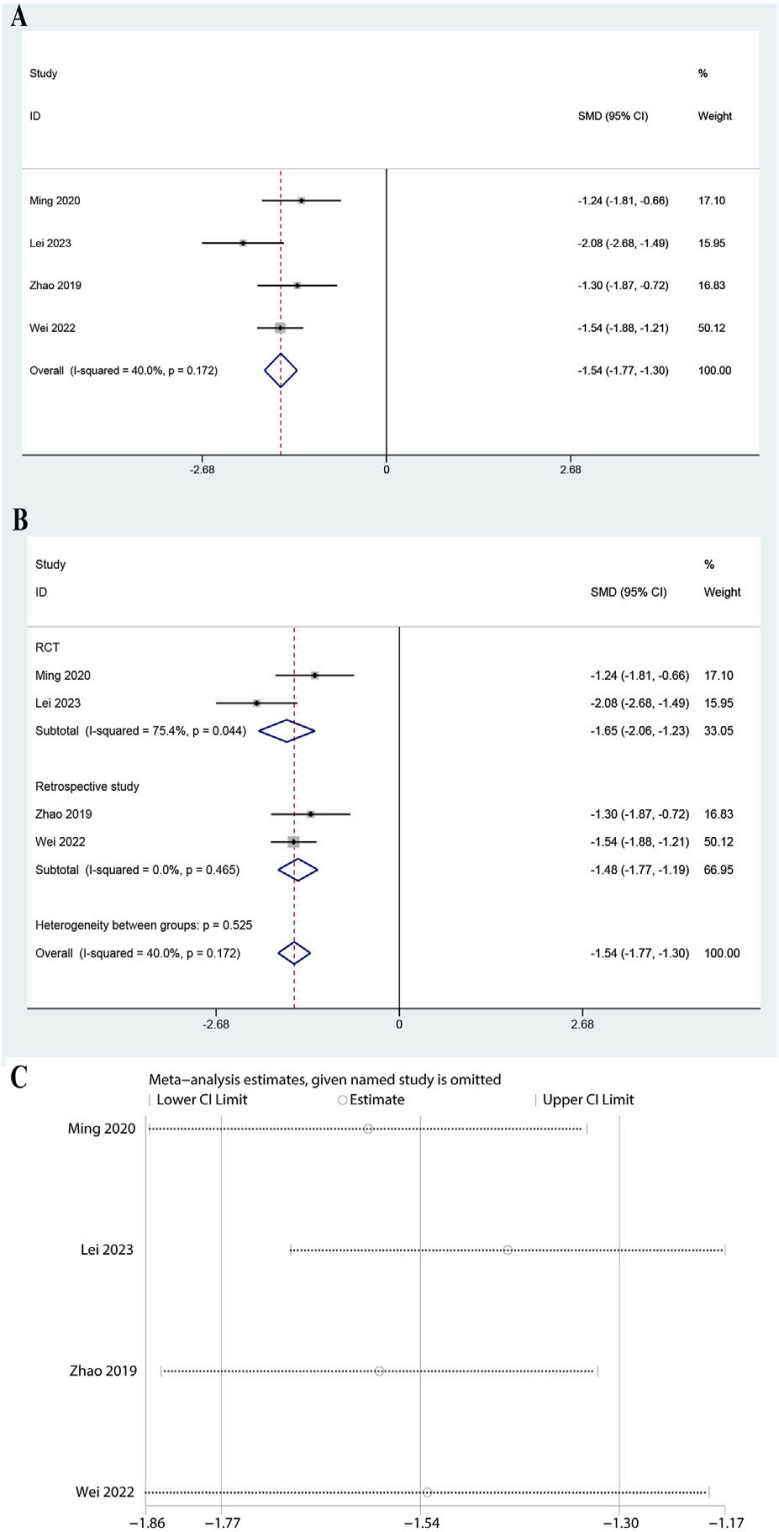
Meta-analysis of Effect of Ulinastatin Combined with Continuous Blood Purification versus Control on Interleukin-1 beta (IL-1b) for Patients with Sepsis. **(A)**: Forest plot displays the pooled standardized mean differences (SMD) with 95% confidence intervals (CI) for the effect. **(B)**: Subgroup analysis shows separate meta-analyses stratified by study design. **(C)**: Sensitivity analysis illustrates leave-one-out analyses to evaluate the influence of individual studies on the overall effect size.

For IL-1β, data from four studies were analyzed, showing a significant reduction (SMD: 1.536, 95% CI: 1.773 to −1.299, P < 0.0001, Figure 3A). The heterogeneity was moderate (I^2^ = 40.0%, P = 0.172), and thus a fixed-effects model was adopted. The RCT subgroup showed an SMD of −1.645 (95% CI: 2.058 to −1.233, P < 0.0001) with high heterogeneity (I^2^ = 75.4%, P = 0.044). The retrospective subgroup showed an SMD of −1.482 (95% CI: 1.771 to −1.192, P < 0.0001) with no heterogeneity (Figure 3B, I^2^ = 0.0%, P = 0.465). Subgroup analysis for doses of ulinastatin showed a significant reduction in both the >200,000 U group (SMD: 1.24, 95% CI: 1.81 to −0.66, P < 0.05) and the ≤200,000 U group (SMD: 1.60, 95% CI: 1.86 to −1.34, I^2^ = 46.4%, P < 0.05, Supplementary Figure S4). The findings of the meta-analysis are reliable, as demonstrated by the stable results across all sensitivity analyses (Figure 3C). These findings suggested that ulinastatin combined with CBP effectively reduces IL-1β levels.

### 3.5 Meta-analysis of IL-6

**FIGURE 4:**
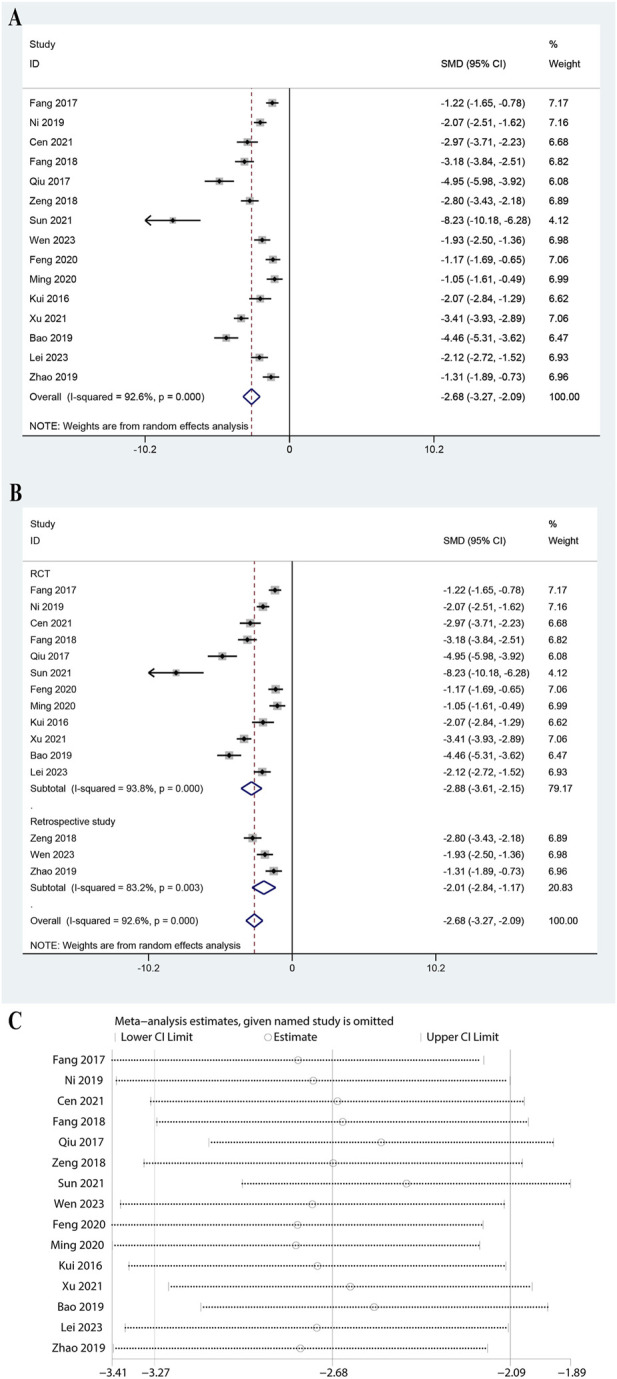
Meta-analysis of Effect of Ulinastatin Combined with Continuous Blood Purification versus Control on Interleukin-6 (IL-6) for Patients with Sepsis. **(A)**: Forest plot displays the pooled standardized mean differences (SMD) with 95% confidence intervals (CI) for the effect. **(B)**: Subgroup analysis shows separate meta-analyses stratified by study design. **(C)**: Sensitivity analysis illustrates leave-one-out analyses to evaluate the influence of individual studies on the overall effect size.

The analysis included data from 15 studies and showed a significant reduction in IL-6 levels with a pooled SMD of −2.679 (95% CI: 3.271 to −2.086, P < 0.0001, Figure 4A). The heterogeneity was high (I^2^ = 92.6%, P = 0.000), necessitating a random-effects model. Subgroup analysis showed significant reductions in both RCTs (SMD: 2.882, 95% CI: 3.612 to −2.152, P < 0.0001, I^2^ = 93.8%) and retrospective studies (SMD: 2.006, 95% CI: 2.839 to −1.174, P < 0.0001, I^2^ = 83.2%, Figure 4B). Subgroup analysis for doses of ulinastatin showed a significant reduction in both the >200,000 U group (SMD: 2.19, 95% CI: 2.97 to −1.42, P < 0.05, I^2^ = 89.7%) and the ≤200,000 U group (SMD: 3.06, 95% CI: 3.94 to −2.18, P < 0.05, I^2^ = 94.1%, Supplementary Figure S5). The robustness of the meta-analysis was confirmed as omitting any study did not lead to significant shifts in the overall outcome (Figure 4C). These results indicated that ulinastatin combined with CBP is effective in lowering IL-6 levels, a critical marker of inflammation in sepsis.

### 3.6 Meta-analysis of IL-8

**FIGURE 5:**
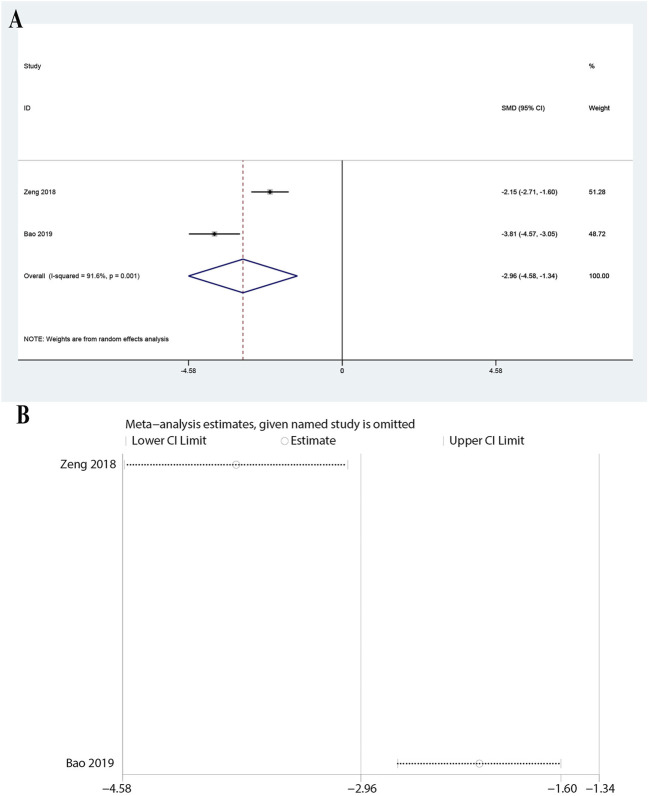
Meta-analysis of Effect of Ulinastatin Combined with Continuous Blood Purification versus Control on Interleukin-8 (IL-8) for Patients with Sepsis. **(A)**: Forest plot displays the pooled standardized mean differences (SMD) with 95% confidence intervals (CI) for the effect. **(B)**: Sensitivity analysis illustrates leave-one-out analyses to evaluate the influence of individual studies on the overall effect size.

Data from two studies showed a significant reduction in IL-8 levels with a pooled SMD of −2.959 (95% CI: 4.582 to −1.337, P < 0.0001). The heterogeneity was high (I^2^ = 91.6%, P = 0.001, Figure 5A), suggesting variability among the studies. Despite this, the significant reduction in IL-8 levels supports the anti-inflammatory effect of ulinastatin combined with CBP in sepsis patients. The sensitivity analysis demonstrated that the overall results remained stable when any single study was omitted from the meta-analysis (Figure 5B).

### 3.7 Meta-analysis of IL-10

**FIGURE 6:**
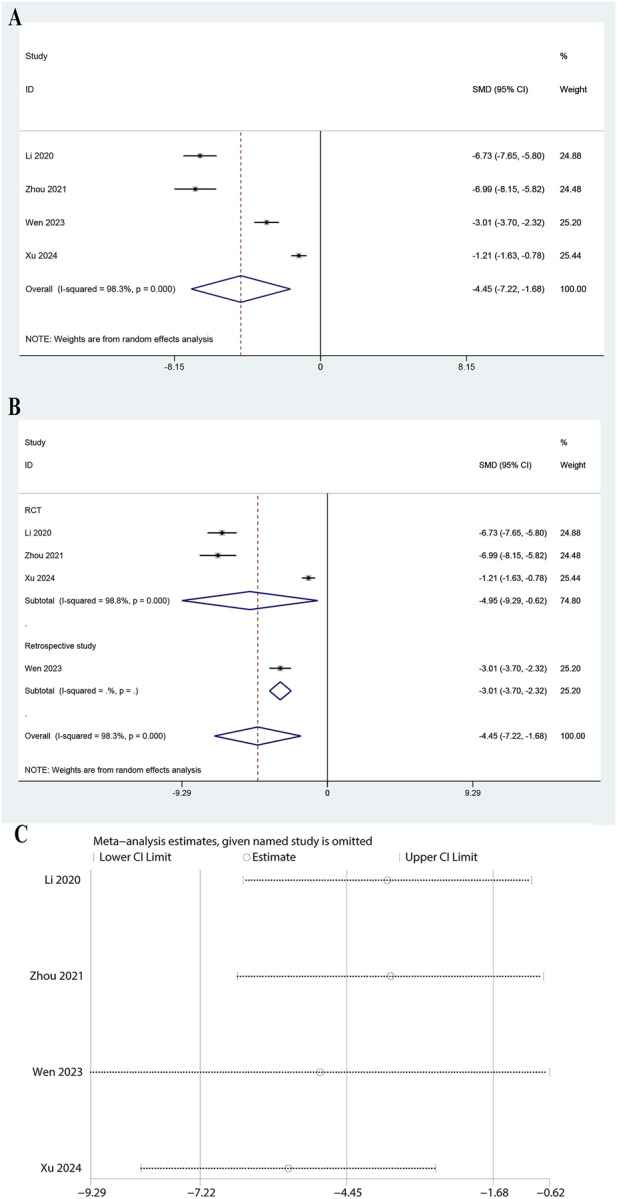
Meta-analysis of Effect of Ulinastatin Combined with Continuous Blood Purification versus Control on Interleukin-10 (IL-10) for Patients with Sepsis. **(A)**: Forest plot displays the pooled standardized mean differences (SMD) with 95% confidence intervals (CI) for the effect. **(B)**: Subgroup analysis shows separate meta-analyses stratified by study design. **(C)**: Sensitivity analysis illustrates leave-one-out analyses to evaluate the influence of individual studies on the overall effect size.

The meta-analysis of four studies indicated a significant reduction in IL-10 levels with a pooled SMD of −4.449 (95% CI: 7.216 to −1.682, P = 0.002, Figure 6A). The heterogeneity was very high (I^2^ = 98.3%, P = 0.000). Subgroup analysis revealed a significant reduction in both RCTs (SMD: 4.952, 95% CI: 9.286 to −0.619, P = 0.025, I^2^ = 98.8%) and retrospective studies (SMD: 3.009, 95% CI: 3.698 to −2.319, P < 0.0001, Figure 6B). The leave-one-out sensitivity analysis showed minimal changes in the confidence intervals, suggesting the results are stable (Figure 6C). These findings suggest that ulinastatin combined with CBP significantly lowers IL-10 levels.

### 3.8 Meta-analysis of PCT

**FIGURE 7:**
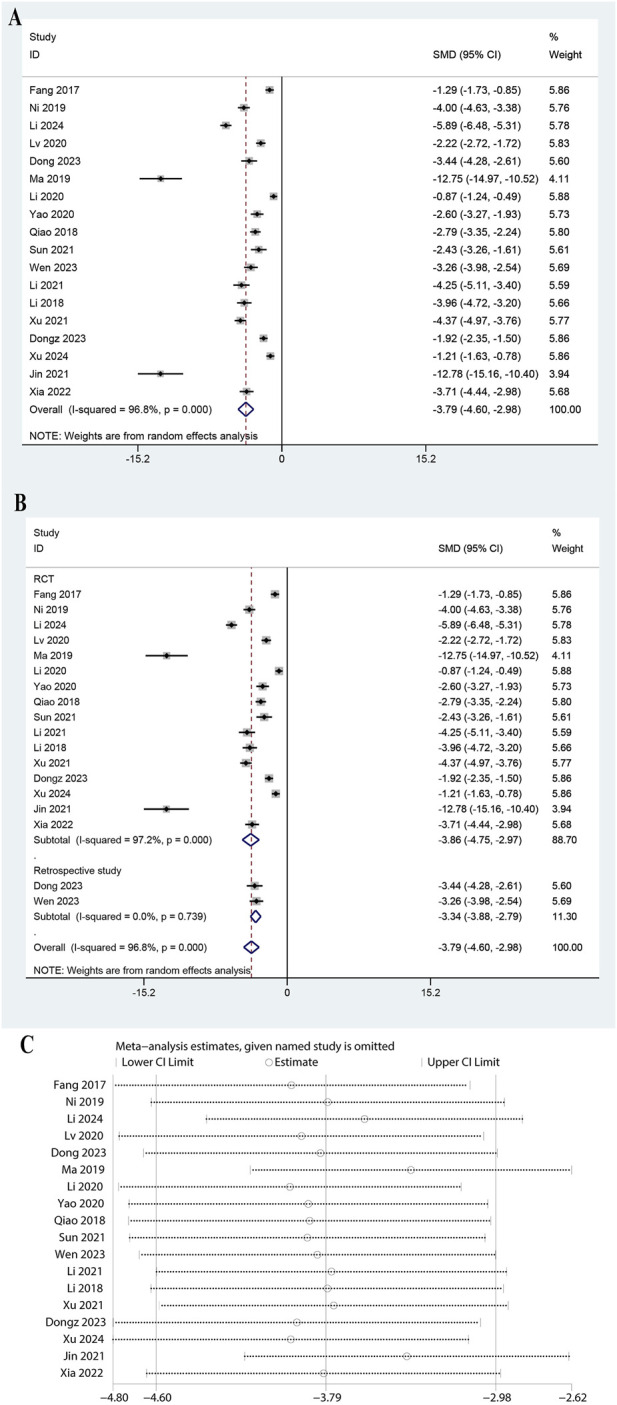
Meta-analysis of Effect of Ulinastatin Combined with Continuous Blood Purification versus Control on Procalcitonin (PCT) for Patients with Sepsis. **(A)**: Forest plot displays the pooled standardized mean differences (SMD) with 95% confidence intervals (CI) for the effect. **(B)**: Subgroup analysis shows separate meta-analyses stratified by study design. **(C)**: Sensitivity analysis illustrates leave-one-out analyses to evaluate the influence of individual studies on the overall effect size.

The meta-analysis included data from 18 studies, showing a significant reduction in PCT levels with a pooled SMD of −3.787 (95% CI: 4.597 to −2.977, P < 0.0001, Figure 7A). The heterogeneity was very high (I^2^ = 96.8%, P = 0.000). Subgroup analysis showed significant reductions in both RCTs (SMD: 3.859, 95% CI: 4.746 to −2.971, P < 0.0001, I^2^ = 97.2%) and retrospective studies (SMD: 3.337, 95% CI: 3.882 to −2.791, P < 0.0001, Figure 7B). Subgroup analysis for doses of ulinastatin showed a significant reduction in both the >200,000 U group (SMD: 1.10, 95% CI: 1.36 to −0.84, P < 0.05, I^2^ = 18.4%) and the ≤200,000 U group (SMD: 4.32, 95% CI: 5.15 to −3.49, P < 0.05, I^2^ = 95.3%, Supplementary Figure S6). The robustness of the meta-analysis was confirmed as omitting any study did not lead to significant shifts in the overall outcome (Figure 7C). These results highlight the efficacy of ulinastatin combined with CBP in reducing PCT levels.

### 3.9 Meta-analysis of TNF-α

**FIGURE 8:**
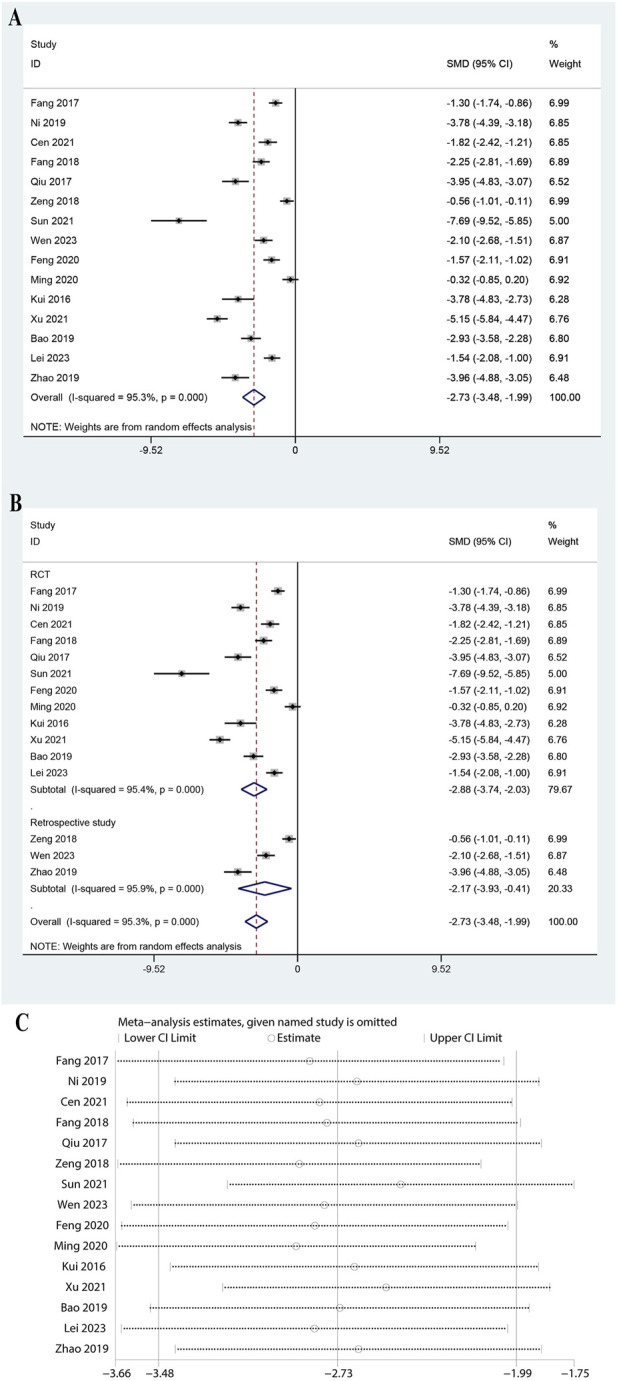
Meta-analysis of Effect of Ulinastatin Combined with Continuous Blood Purification versus Control on Tumor Necrosis Factor-alpha (TNF-a) for Patients with Sepsis. **(A)**: Forest plot displays the pooled standardized mean differences (SMD) with 95% confidence intervals (CI) for the effect. **(B)**: Subgroup analysis shows separate meta-analyses stratified by study design. **(C)**: Sensitivity analysis illustrates leave-one-out analyses to evaluate the influence of individual studies on the overall effect size.

The analysis of 15 studies showed a significant reduction in TNF-α levels with a pooled SMD of −2.734 (95% CI: 3.480 to −1.987, P < 0.0001, Figure 8A). The heterogeneity was high (I^2^ = 95.3%, P = 0.000). Subgroup analysis indicated significant reductions in both RCTs (SMD: 2.883, 95% CI: 3.737 to −2.030, P < 0.0001, I^2^ = 95.4%) and retrospective studies (SMD: 2.169, 95% CI: 3.931 to −0.406, P = 0.016, I^2^ = 95.9%, Figure 8B). Subgroup analysis for doses of ulinastatin showed a significant reduction in both the >200,000 U group (SMD: 1.60, 95% CI: 2.38 to −0.82, P < 0.05, I^2^ = 91.5%) and the ≤200,000 U group (SMD: 3.49, 95% CI: 4.47 to −2.52, P < 0.05, I^2^ = 94.3%, Supplementary Figure S7). Sensitivity analysis revealed that the conclusions were not sensitive to the exclusion of any individual study (Figure 8C). These results suggest that ulinastatin combined with CBP effectively lowers TNF-α.

### 3.10 Meta-analysis of mortality

For the mortality, data from 12 studies were analyzed, showing a significant reduction (OR: 0.30, 95% CI: 0.22 to 0.42, p < 0.0001, Supplementary Figure S8A). There was no observed heterogeneity (I^2^ = 0.0%, p = 0.819), and thus a fixed-effects model was applied. Subgroup analysis for doses of ulinastatin showed a significant reduction in both the >200,000 U group (OR: 0.36, 95% CI: 0.21 to 0.63, P < 0.05, I^2^ = 0.1%) and the ≤200,000 U group (OR: 0.28, 95% CI: 0.18 to 0.42, P < 0.05, I^2^ = 0.0%, Supplementary Figure S8B). The leave-one-out sensitivity analysis showed minimal changes in the confidence intervals, suggesting the results are stable (Supplementary Figure S8C).

### 3.11 Sensitivity analysis and publication bias assessment

The sensitivity analyses further confirmed the robustness of the meta-analysis results, showing that removing each study had little impact on the overall findings (Figures 2C–8C). The funnel plot, which visually inspects asymmetry, suggested the presence of publication bias (Supplementary Figure S9). The results of Egger’s test indicated the presence of significant publication bias, with the bias coefficient being −11.10263 (P = 0.002). The trim-and-fill analysis, however, did not trim any studies, indicating that the results remained unchanged after the analysis, reinforcing the robustness of the findings despite the presence of publication bias.

## 4 Discussion

To the best of our knowledge, this is the first systematic review and meta-analysis reporting on the immunomodulatory effects of ulinastatin in the treatment of sepsis. This meta-analysis demonstrated that ulinastatin significantly reduces mortality and inflammatory markers such as CRP, IL-1β, IL-6, IL-8, IL-10, PCT, and TNF-α in patients with sepsis compared to control group. The reduction was robust across various subgroups for study designs and doses of ulinastatin, although accompanied by high heterogeneity, which suggests variability in study designs, populations, and treatment protocols. The results indicated that ulinastatin effectively modulates the inflammatory response in sepsis, which could potentially translate into clinical benefits such as reduced organ dysfunction and improved outcomes.

Sepsis is associated with an excessive inflammatory response that can damage critical organs including the lungs, liver, kidneys, and cardiovascular system, leading to multiple organ failure and high mortality rates (Zhang and Ning, 2021). CRP is an acute-phase protein that increases significantly in response to inflammation. Elevated CRP levels are associated with sepsis and can be used as a marker to monitor the progression and severity of the infection (Tan et al., 2019). PCT is a precursor of the hormone calcitonin and is produced in response to bacterial infections. It is a highly specific marker for sepsis and can be used to distinguish bacterial infections from other causes of inflammation (Tan et al., 2019). Lv et al. suggested that combining ulinastatin with CBP significantly improve the treatment of sepsis in children by reducing inflammation and lowering CRP and PCT levels (Lv et al., 2020). The meta-analysis showed that ulinastatin combined with CBP significantly reduced CRP and PCT levels compared to the control group. By lowering these biomarkers, the combination therapy not only mitigates the immediate inflammatory response but also potentially reduces the risk of long-term organ damage and improves overall survival rates.

IL-1β is a critical mediator in the pathophysiology of sepsis, significantly contributing to the disease’s progression and severity. This cytokine plays a central role in the early stages of sepsis by initiating and amplifying the inflammatory response. The elevated levels of IL-1β observed in septic patients trigger the release of additional pro-inflammatory mediators, leading to a widespread systemic inflammatory response. This cascade of inflammation not only helps in fighting the infection but also causes substantial collateral damage to the host’s own tissues (Ge et al., 2019). IL-1β induces vasodilation, hypotension, and capillary leakage, which collectively impair tissue perfusion and oxygenation. These vascular changes are detrimental, as they can precipitate multiple organ failure, one of the leading causes of mortality in septic patients. Effective management of sepsis, therefore, often focuses on controlling the levels of IL-1β to mitigate its harmful effects (Yoza and McCall, 2011). By reducing the inflammatory burden and preventing the excessive immune response, such treatments could potentially decrease the incidence of organ failure and increase survival rates in septic patients. This meta-analysis included data from four studies and showed a significant reduction in IL-1β levels. The reduction in IL-1β levels could be associated with a decrease in systemic inflammation, improved hemodynamic stability, and better organ function. Continued research and clinical trials are necessary to refine these therapeutic approaches and confirm their efficacy in broader patient populations. Additionally, the reduction in mortality and inflammatory markers was observed consistently in both the >200,000 U and ≤200,000 U ulinastatin dose groups. This suggests that ulinastatin is effective across a range of dosing regimens. However, the wide variation in doses used across studies, from 100,000 to 500,000 U, highlights the need for further research to determine the optimal dosing strategy. Establishing standardized dosing guidelines will be important to maximize clinical benefits while minimizing potential risks.

The immunomodulatory effects of ulinastatin are mediated through multiple interconnected pathways. Ulinastatin inhibits the NF-κB signaling pathway, a central regulator of pro-inflammatory cytokine production. By suppressing TLR4/MyD88-dependent NF-κB activation, ulinastatin reduces the expression of cytokines such as TNF-α, IL-6, and IL-1β, thereby attenuating systemic inflammation (Cao et al., 2018). Ulinastatin modulates myeloid-derived suppressor cell (MDSC) dynamics. MDSCs, which expand during sepsis to suppress excessive inflammation, can paradoxically contribute to immunosuppression in chronic phases. Ulinastatin reduces pathological MDSC accumulation while promoting their differentiation into immunocompetent myeloid cells, thereby restoring immune homeostasis (Chen et al., 2022).

Based on these findings, ulinastatin should be considered for early use in sepsis patients, with dosing tailored to disease severity to maximize immunomodulatory benefits. Future research must focus on large-scale, multi-center trials across diverse geographic regions to standardize treatment protocols and validate optimal dosing strategies. Additionally, economic evaluations are needed to assess cost-effectiveness, considering both drug costs and potential reductions in ICU stay and mortality. These steps will help guide clinicians in integrating ulinastatin into routine sepsis management more effectively.

This systematic review and meta-analysis have several strengths. First, it provides a comprehensive analysis of multiple inflammatory markers, including CRP, IL-1β, IL-6, IL-8, IL-10, PCT, and TNF-α, which offers a detailed understanding of the immunomodulatory effects of ulinastatin in the treatment of sepsis. Second, the inclusion of a large number of studies with diverse settings enhances the generalizability of the findings, making them applicable to a wide range of clinical scenarios.

This study has several limitations. First, the high heterogeneity observed across the included studies suggests significant variability in study designs, patient populations, and treatment protocols, which may affect the generalizability of the findings. Second, the Egger’s test detected significant publication bias, indicating that smaller studies with less favorable results may be underreported, which could potentially inflate the observed treatment effects and should be considered when interpreting the findings. Third, all the studies included in this meta-analysis were conducted in China, which may limit the applicability of the findings to other geographical regions and populations due to potential differences in healthcare systems, patient demographics, and clinical practices. Fourth, although funding sources were reported in most studies, their potential influence on study outcomes cannot be ruled out and may have affected the results. Fifth, the inclusion of both randomized controlled trials and retrospective studies adds complexity to the analysis, as differences in study design may introduce confounding factors and affect the overall robustness of the pooled results. Finally, while the current evidence is promising, further research is needed to establish the optimal dosing and administration protocols for ulinastatin in combination with CBP. Large-scale, randomized controlled trials should be conducted to confirm these findings and to explore the potential benefits in different patient populations, including those with varying severities of sepsis.

Ulinastatin combined with CBP significantly reduces mortality and levels of various inflammatory markers in sepsis patients, indicating its potential benefit in managing sepsis-related inflammation. However, the high heterogeneity and presence of publication bias highlight the need for further high quality studies to confirm these findings and develop standardized treatment protocols.

## Data Availability

The original contributions presented in the study are included in the article/Supplementary Material, further inquiries can be directed to the corresponding author.
