# Supplementary material for: Immunomodulatory effects of ulinastatin combined with continuous blood purification in sepsis: a systematic review and meta-analysis
Source: Front Pharmacol. 2025 Jul 1;16:1591470. doi: 10.3389/fphar.2025.1591470 (PMC12259711; doi:10.3389/fphar.2025.1591470)
Supplement: Supplementary file 1 [file Table1.docx]

**Search strategy of PubMed**

| NO. | Search Details | Results |
| --- | --- | --- |
| #8 | (#1 OR #2) AND (#3 OR #4) AND (#5 OR #6) Filter: Humans | 570 |
| #7 | (#1 OR #2) AND (#3 OR #4) AND (#5 OR #6) | 674 |
| #6 | ((((((((((((((((((Bloodstream Infection) OR (Bloodstream Infections)) OR (Pyemia)) OR (Pyemias)) OR (Pyohemia)) OR (Pyohemias)) OR (Pyaemia)) OR (Pyaemias)) OR (Septicemia)) OR (Septicemias)) OR (Blood Poisoning)) OR (Blood Poisonings)) OR (Severe Sepsis)) OR (abdominal sepsis)) OR (focal sepsis)) OR (intraabdominal sepsis)) OR (sepsis syndrome)) OR (septic disease)) OR (sepsis) | 360,910 |
| #5 | "Sepsis"[Mesh] | 145,517 |
| #4 | (((((((((((((((Inflammations) OR (Innate Inflammatory Response)) OR (Inflammatory Response, Innate)) OR (Innate Inflammatory Responses)) OR (acute inflammation)) OR (inflammation reaction)) OR (inflammation response)) OR (inflammatory condition)) OR (inflammatory lesion)) OR (inflammatory process)) OR (inflammatory reaction)) OR (inflammatory response)) OR (inflammatory syndrome)) OR (reaction, inflammation)) OR (response, inflammatory)) OR (inflammation) | 1,327,267 |
| #3 | "Inflammation"[Mesh] | 433,379 |
| #2 | ((((((((((UTI68) OR (acid-stable protease inhibitor)) OR (urinary trypsin inhibitor)) OR (urinary trypsin inhibitor-like inhibitor)) OR (UTI)) OR (ulinastatin)) OR (MR 20)) OR (trypsin inhibitor MR-20)) OR (Miraclid)) OR (bikunin)) OR (urinastatin) | 15,198 |
| #1 | "urinastatin" [Supplementary Concept] | 858 |

**Search strategy of EMBASE**

| No. | Query | Results |
| --- | --- | --- |
| #8 | #7 AND 'human'/de | 3215 |
| #7 | (#1 OR #2) AND (#3 OR #4) AND (#5 OR #6) | 3480 |
| #6 | 'bloodstream infection':ti,ab,kw OR 'bloodstream infections':ti,ab,kw OR 'pyemia':ti,ab,kw OR 'pyemias':ti,ab,kw OR 'pyohemia':ti,ab,kw OR 'pyohemias':ti,ab,kw OR 'pyaemia':ti,ab,kw OR 'pyaemias':ti,ab,kw OR 'septicemia':ti,ab,kw OR 'septicemias':ti,ab,kw OR 'blood poisoning':ti,ab,kw OR 'blood poisonings':ti,ab,kw OR 'severe sepsis':ti,ab,kw OR 'abdominal sepsis':ti,ab,kw OR 'focal sepsis':ti,ab,kw OR 'intraabdominal sepsis':ti,ab,kw OR 'sepsis syndrome':ti,ab,kw OR 'septic disease':ti,ab,kw OR 'sepsis':ti,ab,kw | 237592 |
| #5 | 'sepsis'/exp | 359492 |
| #4 | 'inflammations':ti,ab,kw OR 'innate inflammatory response':ti,ab,kw OR 'innate inflammatory responses':ti,ab,kw OR 'acute inflammation':ti,ab,kw OR 'inflammation reaction':ti,ab,kw OR 'inflammation response':ti,ab,kw OR 'inflammatory condition':ti,ab,kw OR 'inflammatory lesion':ti,ab,kw OR 'inflammatory process':ti,ab,kw OR 'inflammatory reaction':ti,ab,kw OR 'inflammatory response':ti,ab,kw OR 'inflammatory syndrome':ti,ab,kw OR 'inflammation':ti,ab,kw | 1070740 |
| #3 | 'inflammation'/exp | 4577112 |
| #2 | 'uti68':ti,ab,kw OR 'acid-stable protease inhibitor':ti,ab,kw OR 'urinary trypsin inhibitor':ti,ab,kw OR 'urinary trypsin inhibitor-like inhibitor':ti,ab,kw OR 'uti':ti,ab,kw OR 'ulinastatin':ti,ab,kw OR 'mr 20':ti,ab,kw OR 'trypsin inhibitor mr-20':ti,ab,kw OR 'miraclid':ti,ab,kw OR 'bikunin':ti,ab,kw OR 'urinastatin':ti,ab,kw | 27581 |
| #1 | 'ulinastatin'/exp | 2261 |

**Search strategy of Cochrane Library**

| NO. | Search deatiles | Hits |
| --- | --- | --- |
| #1 | (UTI68):ti,ab,kw OR (acid-stable protease inhibitor):ti,ab,kw OR (urinary trypsin inhibitor):ti,ab,kw OR (urinary trypsin inhibitor-like inhibitor):ti,ab,kw OR (UTI):ti,ab,kw OR (ulinastatin):ti,ab,kw OR (MR 20):ti,ab,kw OR (trypsin inhibitor MR-20):ti,ab,kw OR (Miraclid):ti,ab,kw OR (bikunin):ti,ab,kw OR (urinastatin):ti,ab,kw | 4509 |
| #2 | MeSH descriptor: [Inflammation] explode all trees | 16457 |
| #3 | (Inflammations):ti,ab,kw OR (Innate Inflammatory Response):ti,ab,kw OR (Innate Inflammatory Responses):ti,ab,kw OR (acute inflammation):ti,ab,kw OR (inflammation reaction):ti,ab,kw OR (inflammation response):ti,ab,kw OR (inflammatory condition):ti,ab,kw OR (inflammatory lesion):ti,ab,kw OR (inflammatory process):ti,ab,kw OR (inflammatory reaction):ti,ab,kw OR (inflammatory response):ti,ab,kw OR (inflammatory syndrome):ti,ab,kw OR (inflammation):ti,ab,kw | 84208 |
| #4 | MeSH descriptor: [Sepsis] explode all trees | 6508 |
| #5 | (Bloodstream Infection):ti,ab,kw OR (Bloodstream Infections):ti,ab,kw OR (Pyemia):ti,ab,kw OR (Pyemias):ti,ab,kw OR (Pyohemia):ti,ab,kw OR (Pyohemias):ti,ab,kw OR (Pyaemia):ti,ab,kw OR (Pyaemias):ti,ab,kw OR (Septicemia):ti,ab,kw OR (Septicemias):ti,ab,kw OR (Blood Poisoning):ti,ab,kw OR (Blood Poisonings):ti,ab,kw OR (Severe Sepsis):ti,ab,kw OR (abdominal sepsis):ti,ab,kw OR (focal sepsis):ti,ab,kw OR (intraabdominal sepsis):ti,ab,kw OR (sepsis syndrome):ti,ab,kw OR (septic disease):ti,ab,kw OR (sepsis):ti,ab,kw | 17585 |
| #6 | #1 and (#2 or #3) and (#4 or #5) | 86 |

**Search strategy of Web of science**

| NO. | Search deatiles | Hits |
| --- | --- | --- |
| #1 | (((((((((TS=(UTI68) OR TS=(acid-stable protease inhibitor)) OR TS=(urinary trypsin inhibitor)) OR TS=(urinary trypsin inhibitor-like inhibitor)) OR TS=(UTI)) OR TS=(ulinastatin)) OR TS=(MR 20)) OR TS=(trypsin inhibitor MR-20)) OR TS=(Miraclid)) OR TS=(bikunin)) OR TS=(urinastatin) | 33202 |
| #2 | (((((((((((TS=(Inflammations) OR TS=(Innate Inflammatory Response)) OR TS=(Innate Inflammatory Responses)) OR TS=(acute inflammation)) OR TS=(inflammation reaction)) OR TS=(inflammation response)) OR TS=(inflammatory condition)) OR TS=(inflammatory lesion)) OR TS=(inflammatory process)) OR TS=(inflammatory reaction)) OR TS=(inflammatory response)) OR TS=(inflammatory syndrome)) OR TS=(inflammation) | 1180203 |
| #3 | (((((((((((((((((TS=(Bloodstream Infection) OR TS=(Bloodstream Infections)) OR TS=(Pyemia)) OR TS=(Pyemias)) OR TS=(Pyohemia)) OR TS=(Pyohemias)) OR TS=(Pyaemia)) OR TS=(Pyaemias)) OR TS=(Septicemia)) OR TS=(Septicemias)) OR TS=(Blood Poisoning)) OR TS=(Blood Poisonings)) OR TS=(Severe Sepsis)) OR TS=(abdominal sepsis)) OR TS=(focal sepsis)) OR TS=(intraabdominal sepsis)) OR TS=(sepsis syndrome)) OR TS=(septic disease)) OR TS=(sepsis) | 196715 |
| #4 | #3 AND #2 AND #1 | 196 |

CNKI

(SU %= 'nongduxing' OR SU %= 'yanzhong nongduxing' OR SU %= 'nongduxing xiuke') AND

(SU %= 'wusitading' OR SU %= 'wusitading zhusheye') AND

(SU %= 'xueye jinghua' OR SU %= 'xueye jinghua liaofa' OR SU %= 'xueye jinghua jishu') AND

(SU %= 'yanzheng' OR SU %= 'yanzheng yinzhi' OR SU %= 'xueqing yanzheng yinzhi' OR SU %= 'yanzheng zhibiao' OR SU %= 'manxing yanzheng' OR SU %= 'jixing yanzheng')

16

WanFang

(zhuti:(nongduxing) OR quanbu:(yanzhong nongduxing) OR zhuti:(nongduxing xiuke)) AND

(zhuti:(wusitading) OR zhuti:(wusitading zhusheye)) AND

(zhuti:(xueye jinghua) OR zhuti:(xueye jinghua liaofa) OR zhuti:(xueye jinghua jishu)) AND

(zhuti:(yanzheng) OR zhuti:(yanzheng yinzhi) OR zhuti:(xueqing yanzheng yinzhi) OR zhuti:(yanzheng zhibiao) OR zhuti:(manxing yanzheng) OR zhuti:(jixing yanzheng))

23

Sinomed

("nongduxing"[changyong ziduan: zhineng] OR "yanzhong nongduxing"[changyong ziduan: zhineng] OR "nongduxing xiuke"[changyong ziduan: zhineng]) AND

("wusitading"[changyong ziduan: zhineng] OR "wusitading zhusheye"[changyong ziduan: zhineng]) AND

("xueye jinghua"[changyong ziduan: zhineng] OR "xueye jinghua liaofa"[changyong ziduan: zhineng] OR "xueye jinghua jishu"[changyong ziduan: zhineng]) AND

("yanzheng"[changyong ziduan: zhineng] OR "yanzheng yinzhi"[changyong ziduan: zhineng] OR "xueqing yanzheng yinzhi"[changyong ziduan: zhineng] OR "yanzheng zhibiao"[changyong ziduan: zhineng] OR "manxing yanzheng"[changyong ziduan: zhineng] OR "jixing yanzheng"[changyong ziduan: zhineng])

67
