# Supplementary figures and images for: Immunomodulatory effects of ulinastatin combined with continuous blood purification in sepsis: a systematic review and meta-analysis
Source: Front Pharmacol. 2025 Jul 1;16:1591470. doi: 10.3389/fphar.2025.1591470 (PMC12259711; doi:10.3389/fphar.2025.1591470)

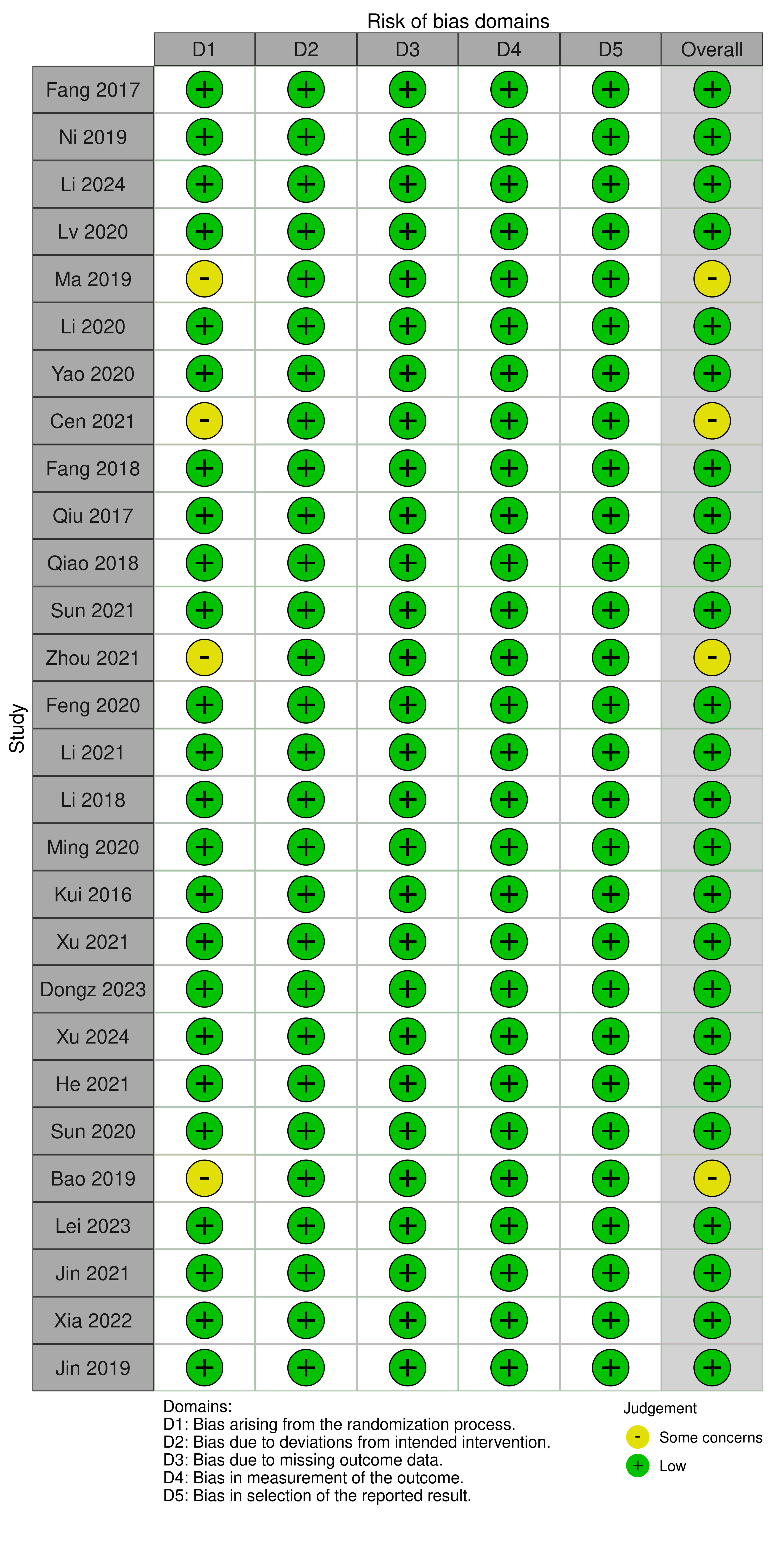

Supplement: Supplementary file 2 [file DataSheet1.zip › Supplementary images(1-9)/Supplemental Figure 1.jpg]

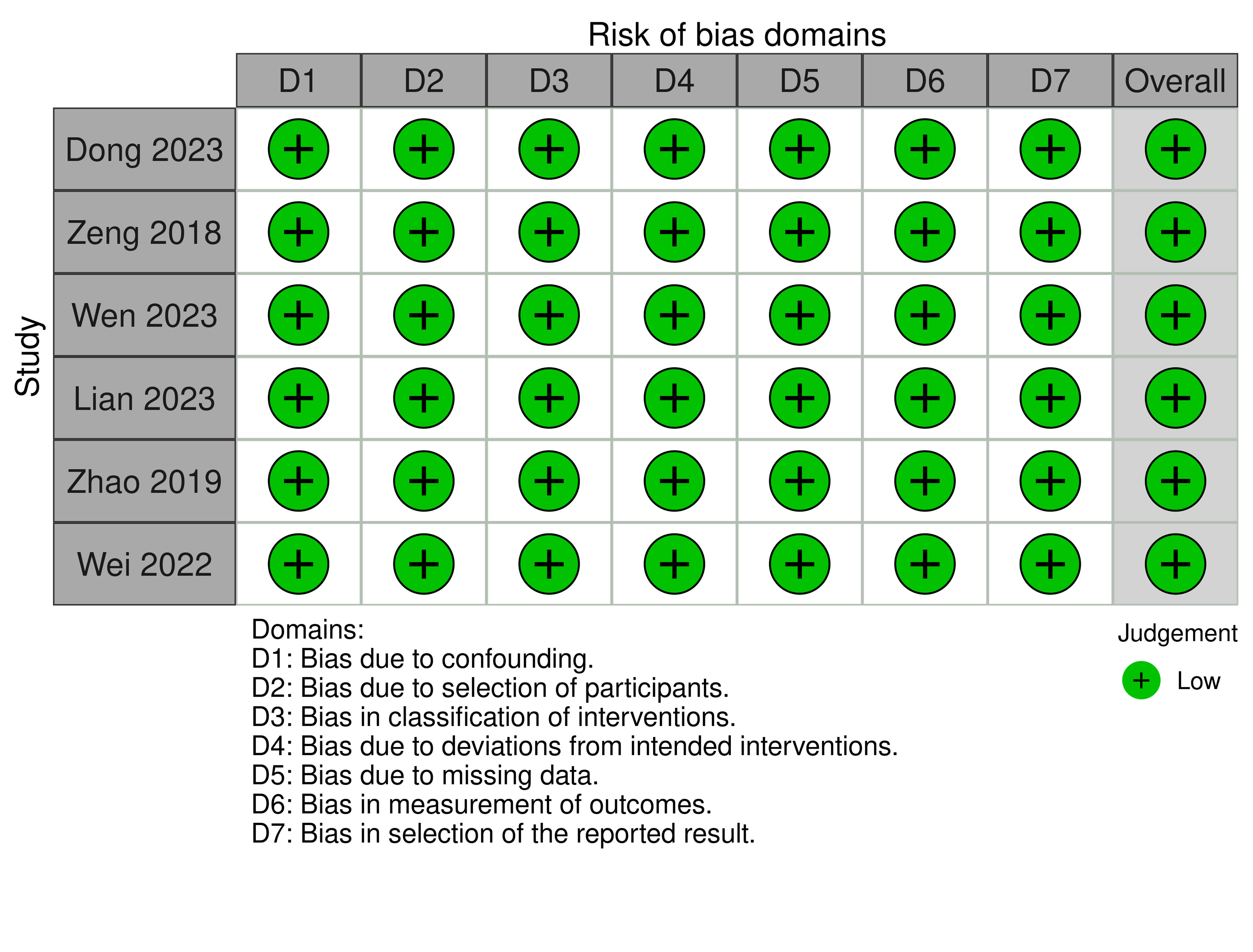

Supplement: Supplementary file 2 [file DataSheet1.zip › Supplementary images(1-9)/Supplemental Figure 2.jpg]

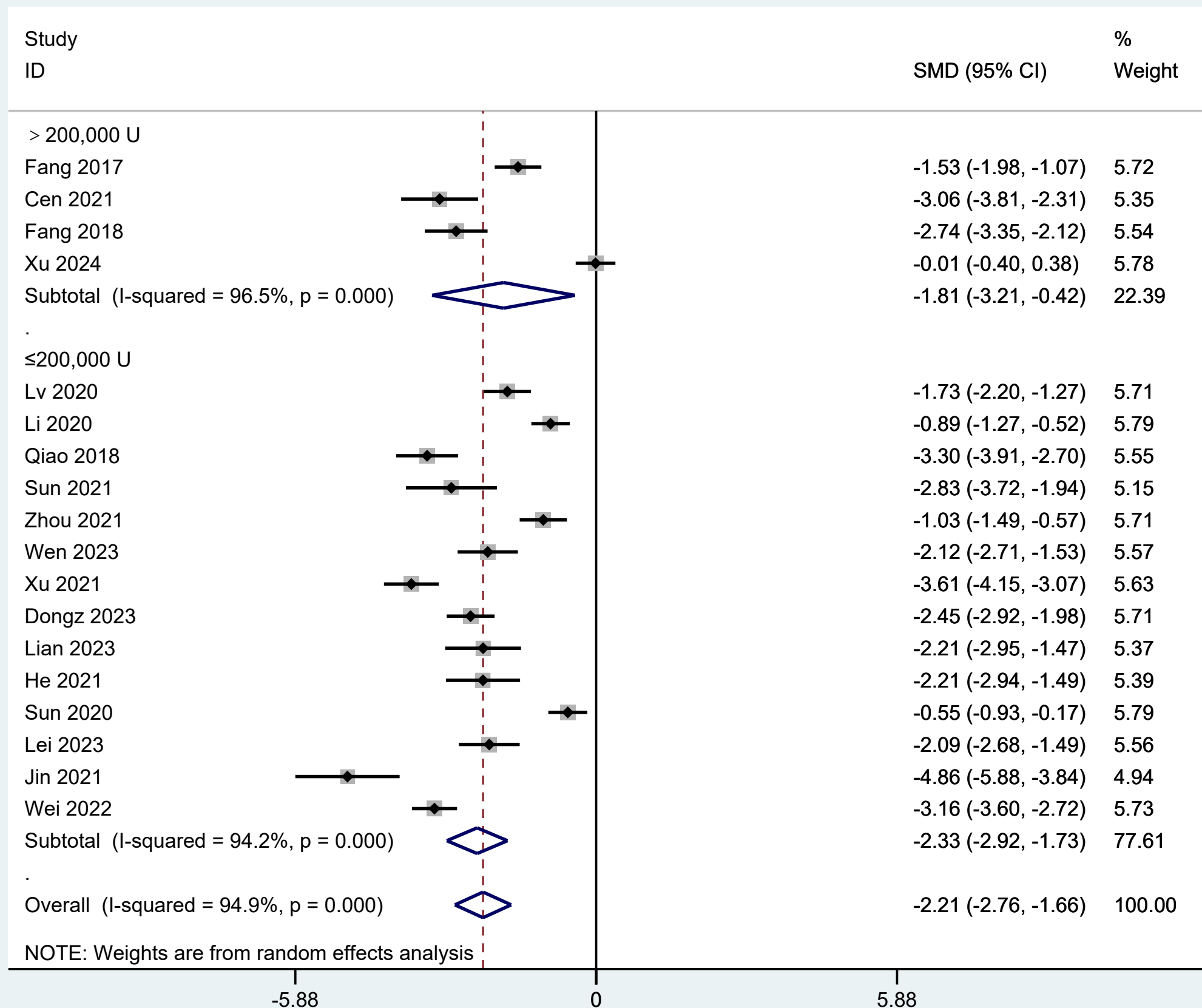

Supplement: Supplementary file 2 [file DataSheet1.zip › Supplementary images(1-9)/Supplemental Figure 3.pdf]

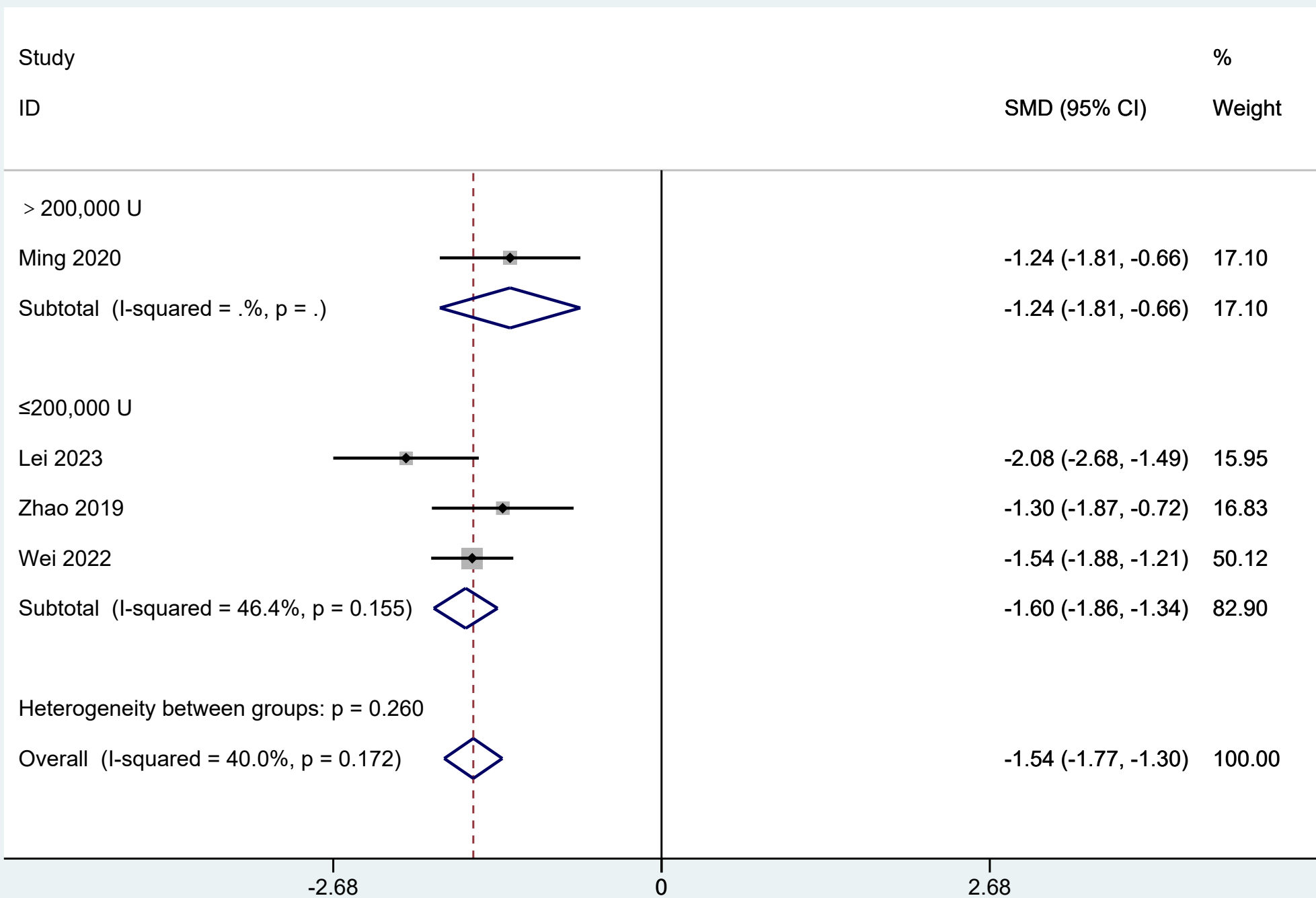

Supplement: Supplementary file 2 [file DataSheet1.zip › Supplementary images(1-9)/Supplemental Figure 4.pdf]

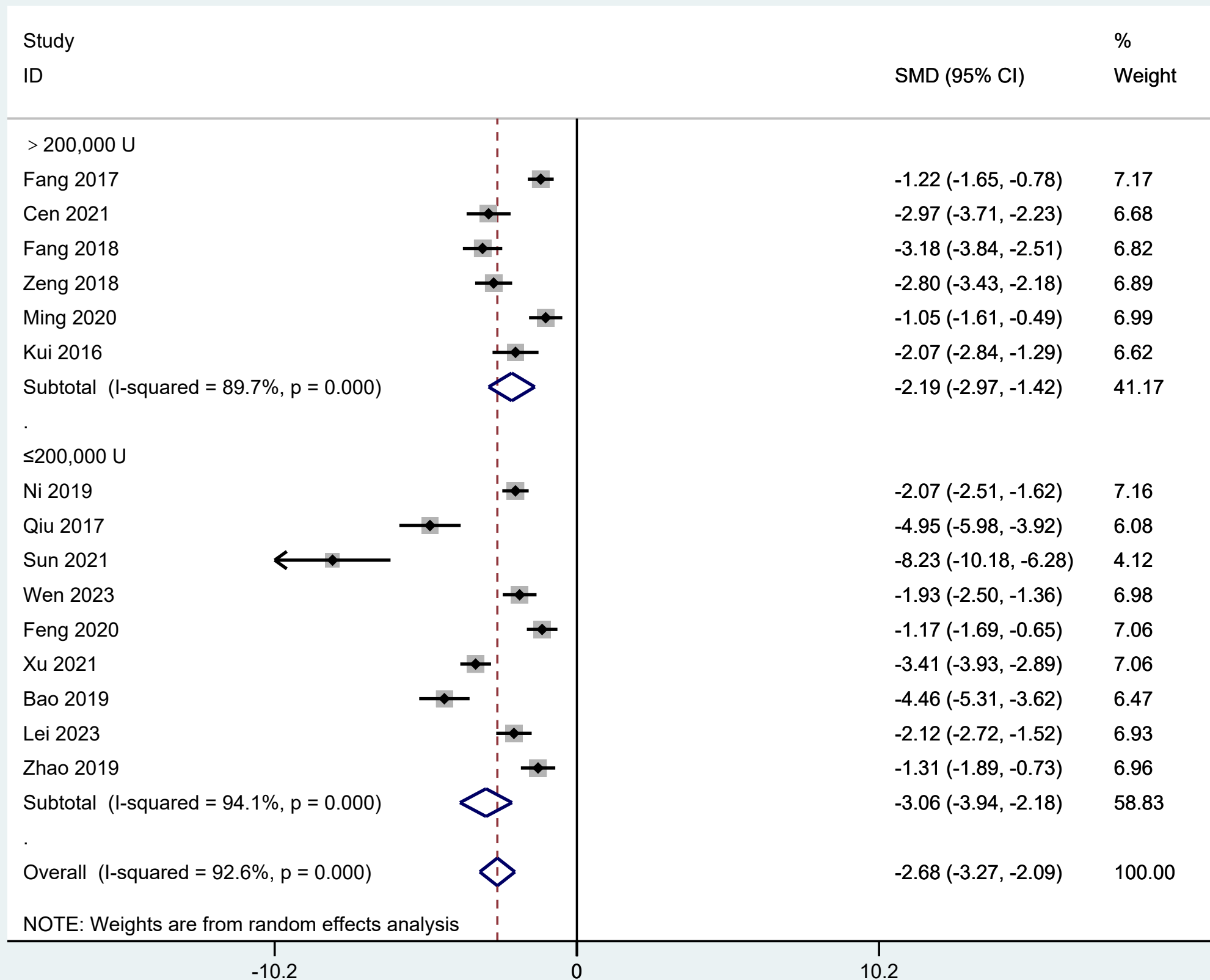

Supplement: Supplementary file 2 [file DataSheet1.zip › Supplementary images(1-9)/Supplemental Figure 5.pdf]

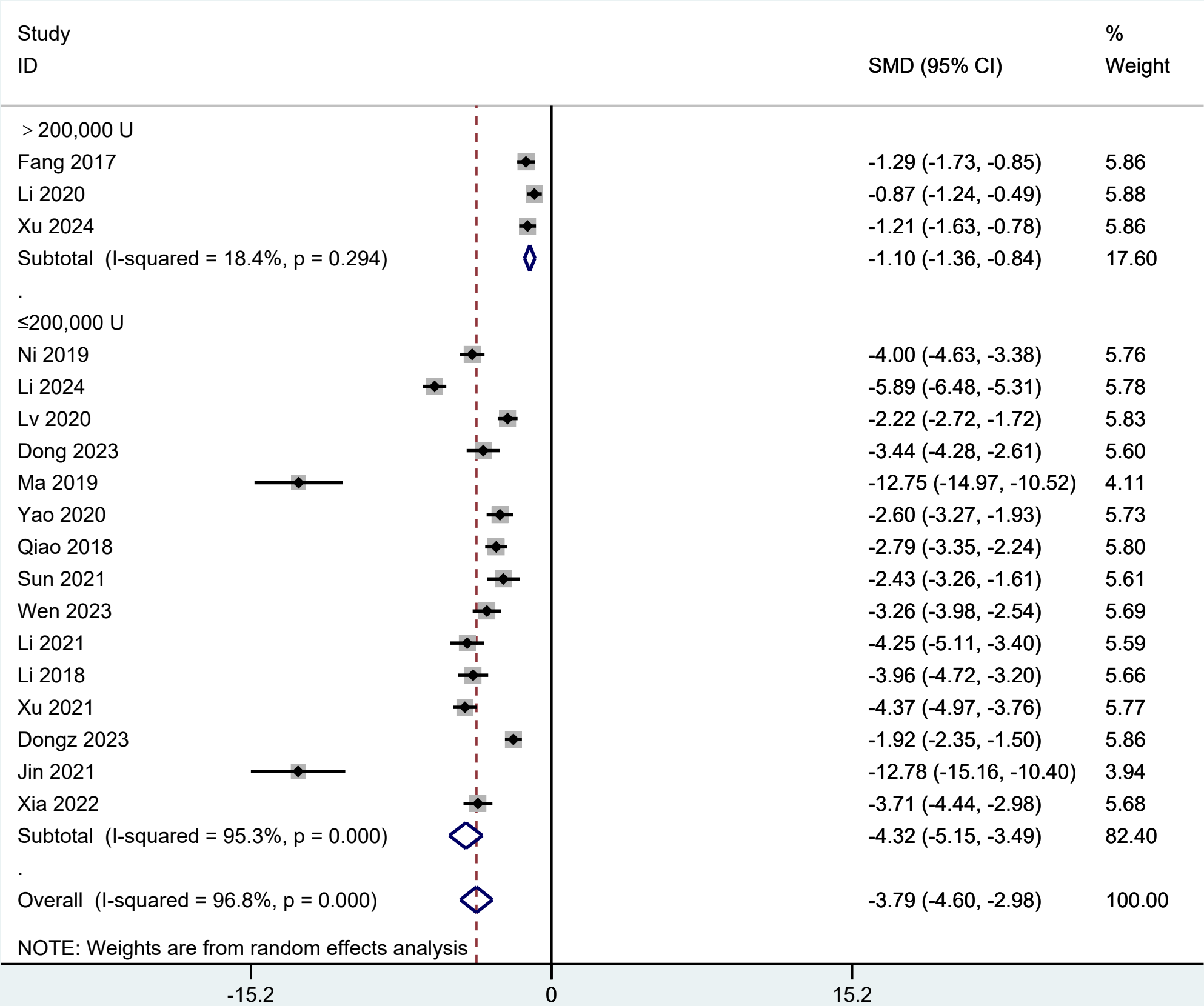

Supplement: Supplementary file 2 [file DataSheet1.zip › Supplementary images(1-9)/Supplemental Figure 6.pdf]

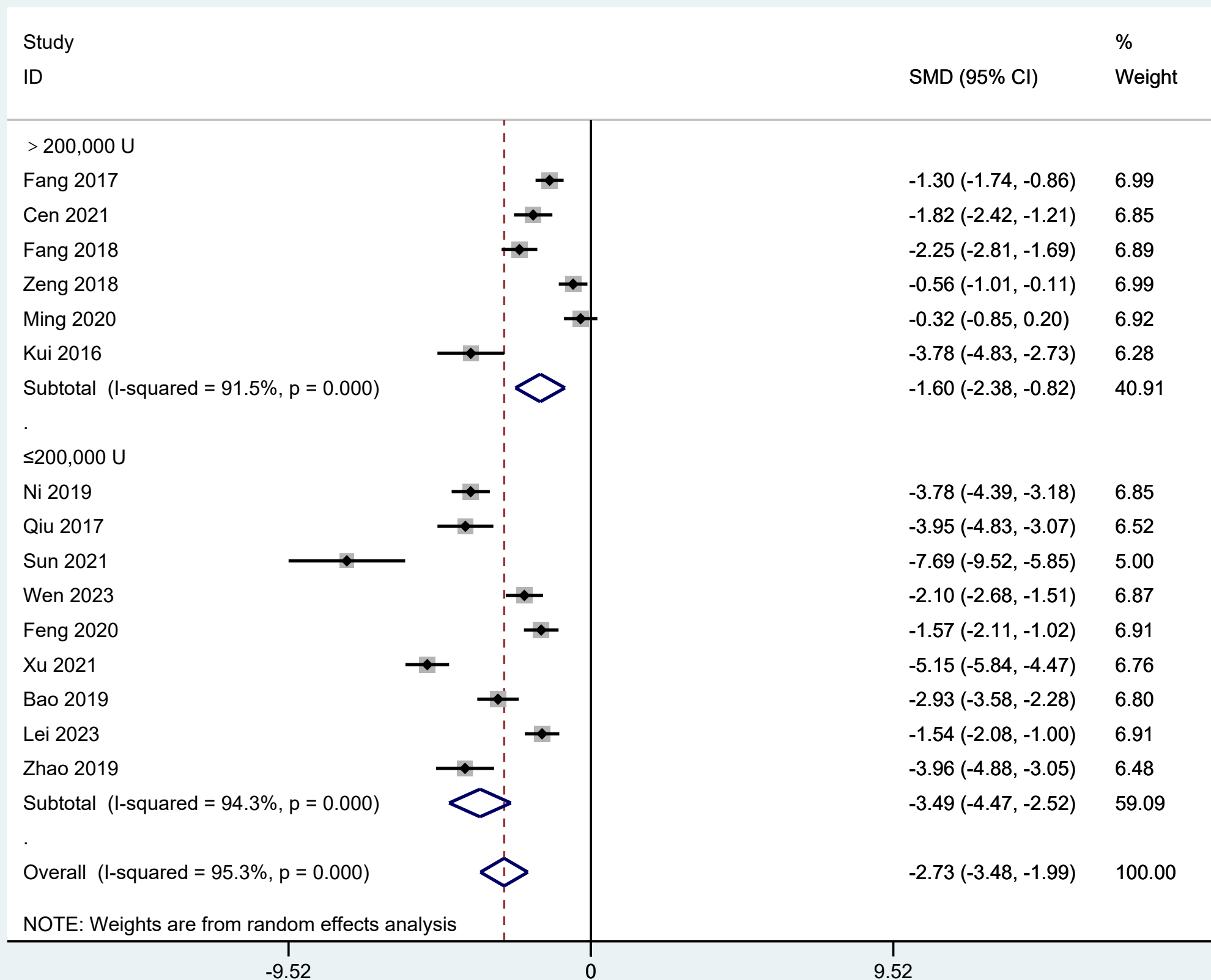

Supplement: Supplementary file 2 [file DataSheet1.zip › Supplementary images(1-9)/Supplemental Figure 7.pdf]

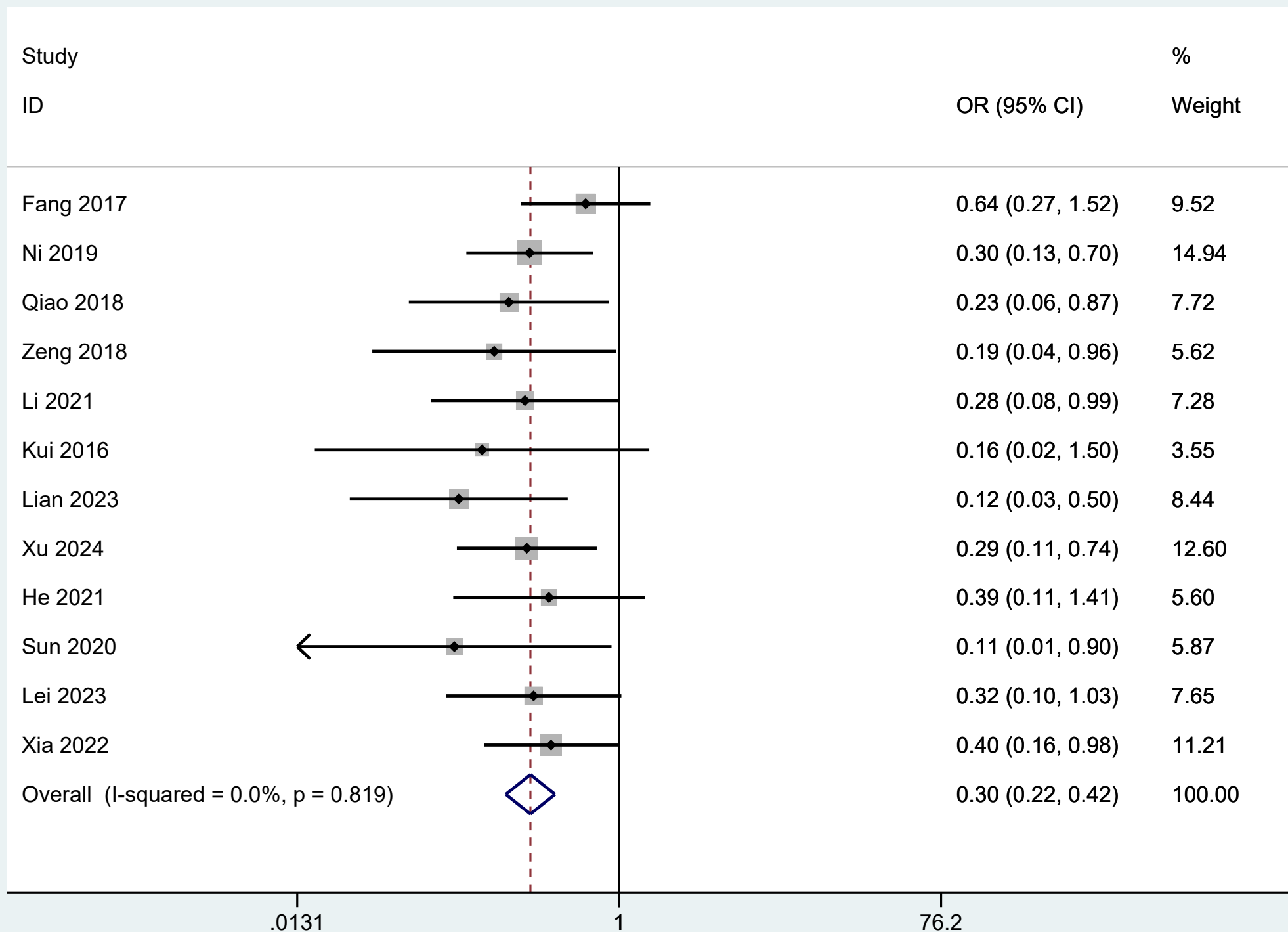

Supplement: Supplementary file 2 [file DataSheet1.zip › Supplementary images(1-9)/Supplemental Figure 8A.pdf]

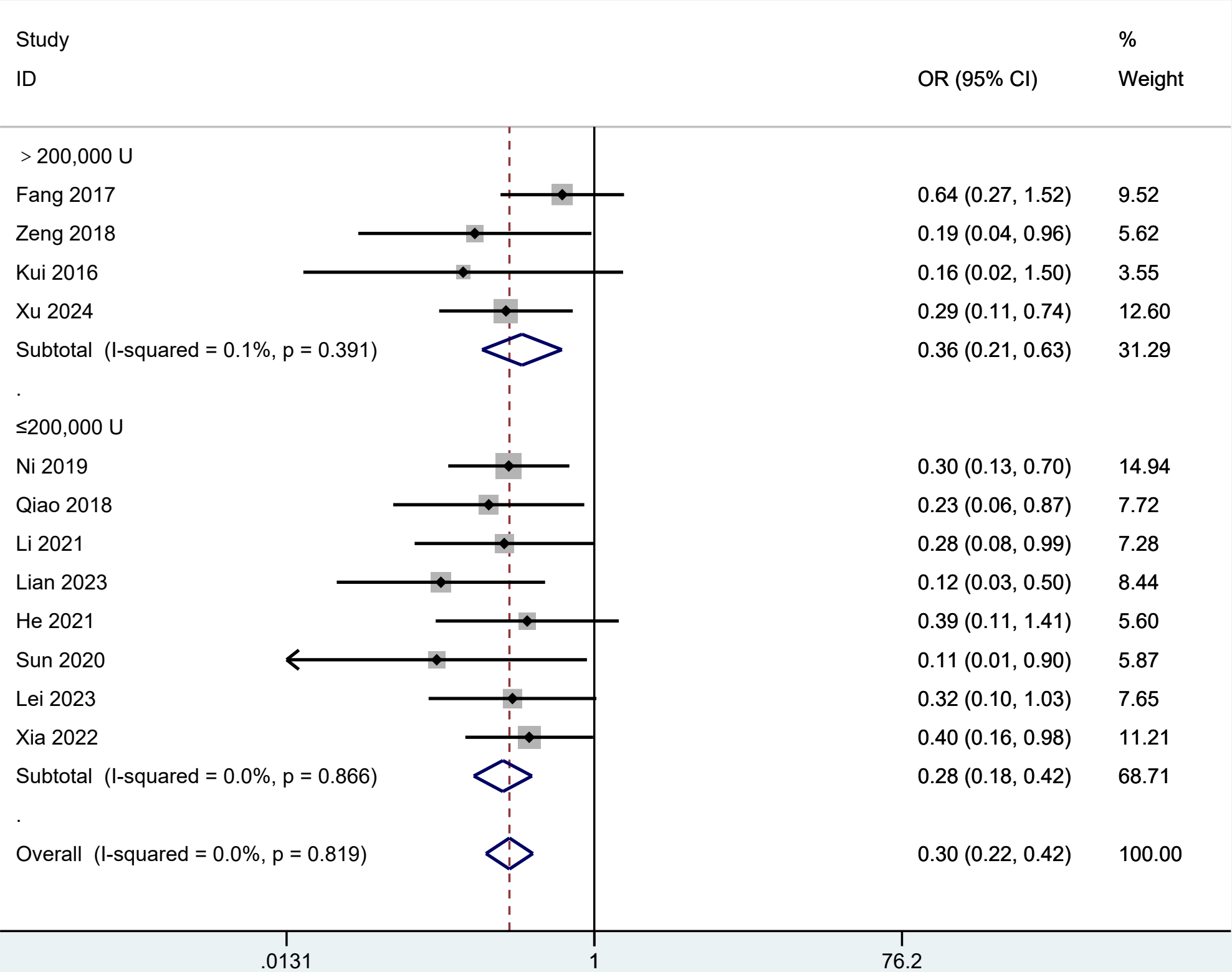

Supplement: Supplementary file 2 [file DataSheet1.zip › Supplementary images(1-9)/Supplemental Figure 8B.pdf]

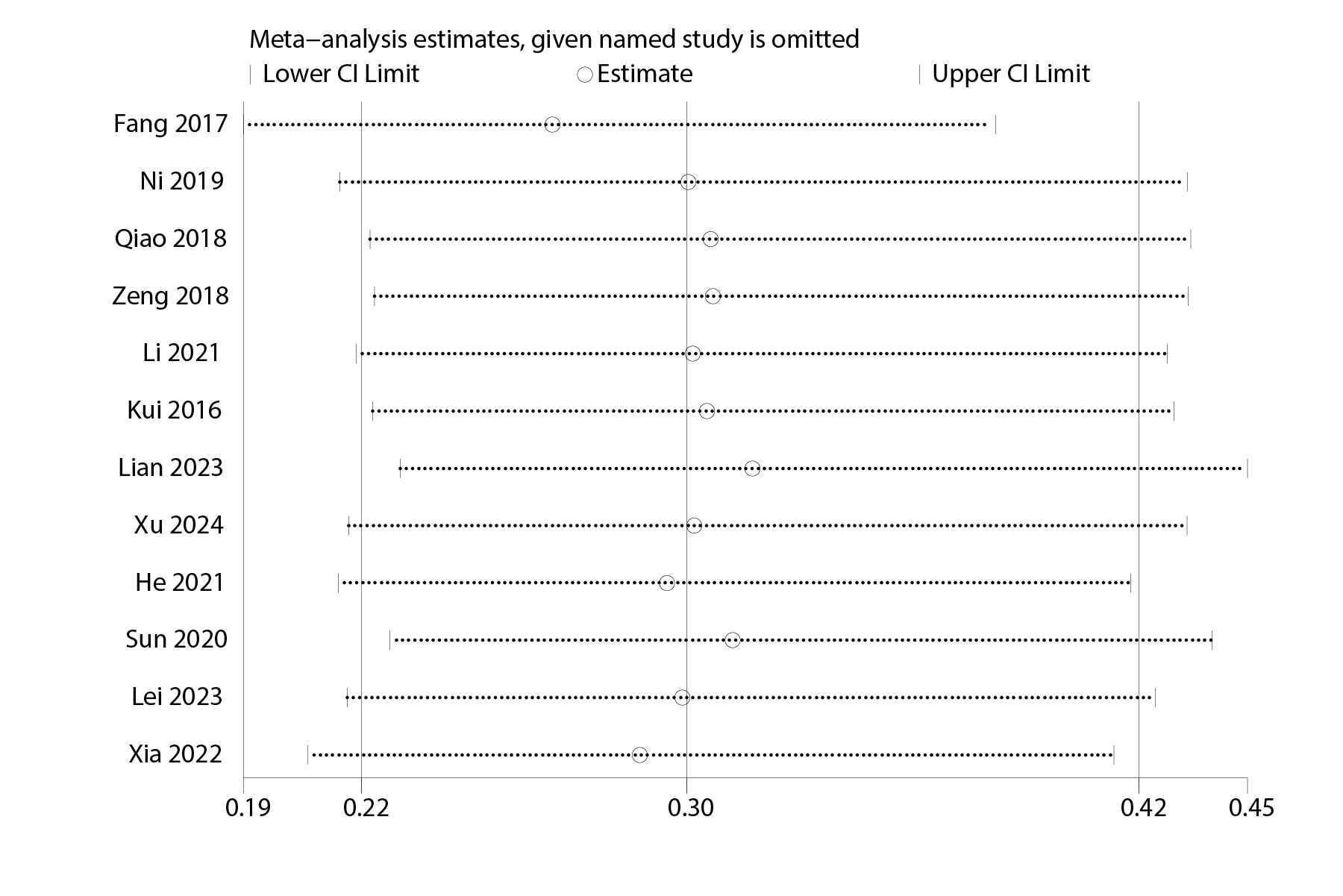

Supplement: Supplementary file 2 [file DataSheet1.zip › Supplementary images(1-9)/Supplemental Figure 8C.png]

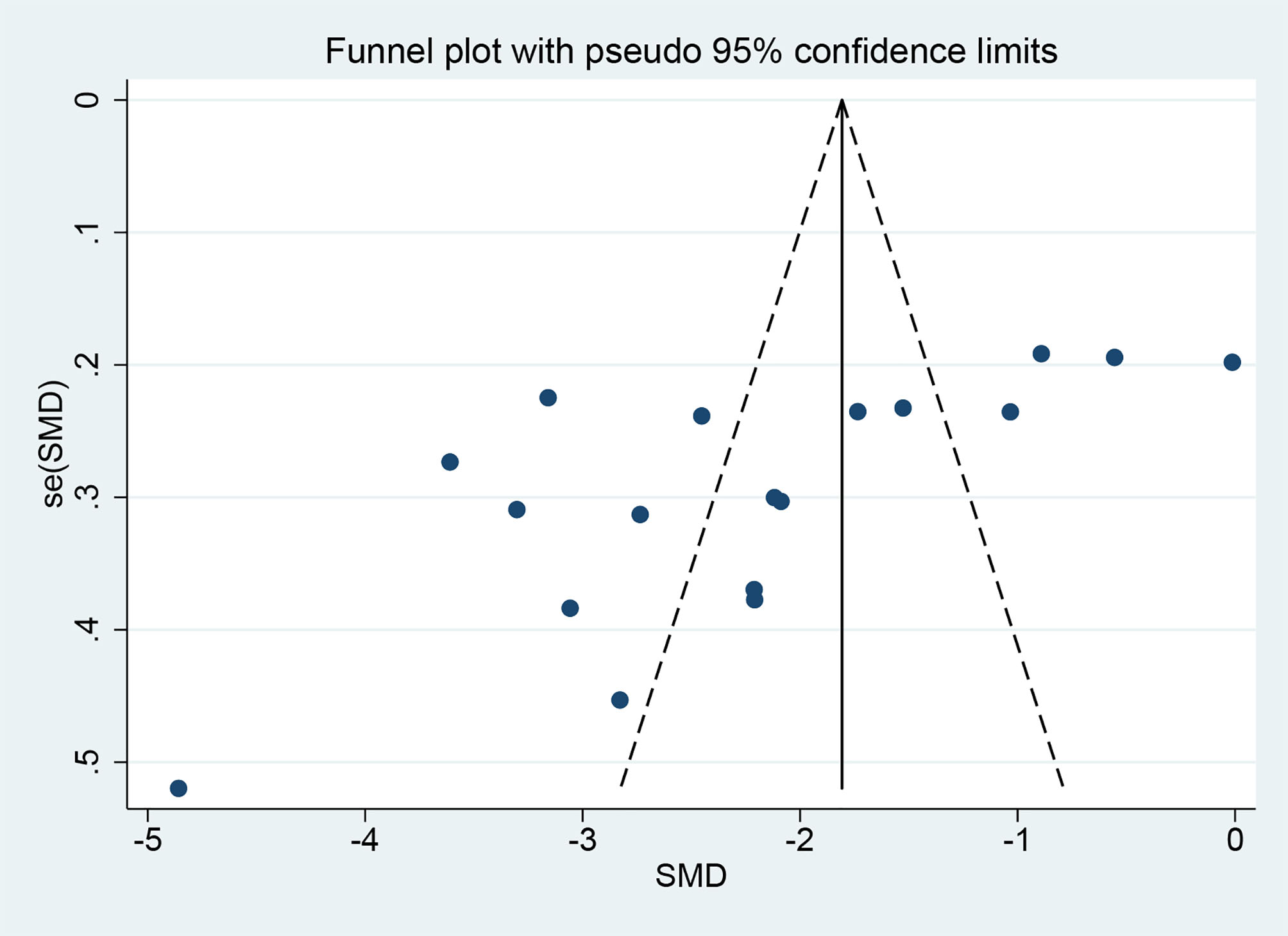

Supplement: Supplementary file 2 [file DataSheet1.zip › Supplementary images(1-9)/Supplemental Figure 9.jpg]
